# Modular design of bi- and multi-specific knob domain fusions

**DOI:** 10.3389/fimmu.2024.1384467

**Published:** 2024-03-27

**Authors:** Mikhail Kuravsky, Glyn F. Gibbons, Callum Joyce, Anthony Scott-Tucker, Alex Macpherson, Alastair D. G. Lawson

**Affiliations:** UK Research, UCB Biopharma UK, Slough, United Kingdom

**Keywords:** bovine antibodies, knob domain, recombinant expression, coiled coil, bispecific

## Abstract

**Introduction:**

The therapeutic potential of bispecific antibodies is becoming widely recognised, with over a hundred formats already described. For many applications, enhanced tissue penetration is sought, so bispecifics with low molecular weight may offer a route to enhanced potency. Here we report the design of bi- and tri-specific antibody-based constructs with molecular weights as low as 14.5 and 22 kDa respectively.

**Methods:**

Autonomous bovine ultra-long CDR H3 (knob domain peptide) modules have been engineered with artificial coiled-coil stalks derived from Sin Nombre orthohantavirus nucleocapsid protein and human Beclin-1, and joined in series to produce bi- and tri-specific antibody-based constructs with exceptionally low molecular weights.

**Results:**

Knob domain peptides with coiled-coil stalks retain high, independent antigen binding affinity, exhibit exceptional levels of thermal stability, and can be readily joined head-to-tail yielding the smallest described multi-specific antibody format. The resulting constructs are able to bind simultaneously to all their targets with no interference.

**Discussion:**

Compared to existing bispecific formats, the reduced molecular weight of the knob domain fusions may enable enhanced tissue penetration and facilitate binding to cryptic epitopes that are inaccessible to conventional antibodies. Furthermore, they can be easily produced at high yield as recombinant products and are free from the heavy-light chain mispairing issue. Taken together, our approach offers an efficient route to modular construction of minimalistic bi- and multi-specifics, thereby further broadening the therapeutic scope for knob domain peptides.

## 1 Introduction

Over the past decade, monoclonal antibodies (mAbs) have experienced explosive growth as therapeutics (1, 2) and have become one of the best-selling modalities of drugs in the market (3). Among the properties that have contributed to the success of mAbs are their exquisite target selectivity combined with their ability to be generated against a broad range of antigens (4, 5). One of the rapidly expanding areas of antibody technology is the development of bispecific constructs (bsAbs) capable of simultaneous binding to multiple targets. Indeed, eight bsAbs won FDA approval in the 2022-2023 period, compared to only three approved before 2022 (2, 6, 7). While the majority of bsAbs were designed as T cell recruiters for cancer treatments (8), they also have a broad range of applications outside the cancer area, including dual targeting (9, 10), localised payload delivery (9), receptor crosslinking (11), restoring protein-protein interactions (12) and potent high-avidity binding (13).

A typical bsAb consists of two heavy chains with different antigen specificities, each paired to its own light chain. Such a design suffers from manufacturability issues precipitated by the unbalanced expression of chains and combinatorial nature of their association (14–16). Several strategies have been proposed to circumvent these issues, including the “knobs-into-holes” (17) and “CrossMab” (18) approaches, as well as improved purification techniques (19). An alternative to the conventional bsAb design is fusion of antibody fragments by means of genetic linking (14–16). More than 100 of fused formats have been developed so far (16), ranging from grafts of additional Fab arms onto full-length IgG (20, 21) to minimalistic variable domain-only designs, such as the BiTE (22) and the diabody formats (23). The single-domain camelid- and shark-derived antibody fragments (VHH and VNAR) can be also used to generate bispecific constructs, by either linking to conventional antibody fragments (24) or joining head-to-tail (13, 25–27). However, despite overcoming manufacturability limitations of the conventional bsAb design, the antibody fusions bring their own set of issues, including increased aggregation tendency (28) and steric obstruction of binding site by a fusion partner (26, 29).

**Figure 1:**
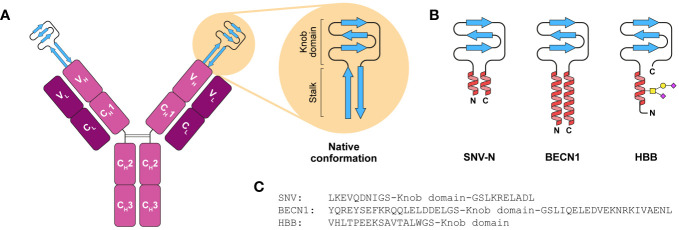
Knob domain expression constructs for high-yield recombinant production in mammalian cells. **(A)** Schematic representation of bovine IgG with an ultra-long CDR H3 region. Knob domain at the tip of the V_H_ region is supported by a β-ribbon stalk. **(B)** Schematic representation of the designed SNV-N, BECN1 and HBB knob domain fusions. The native stalk is substituted with either an antiparallel coiled-coil dimer (SNV-N, BECN1) or a single N-terminal α-helix (HBB) loosely attached to the knob domain via flexible GS linkers. The HBB helix is *O*-glycosylated at one of three potential sites. **(C)** Amino acid sequences of knob domain fusions.

Knob domain peptides are derived from a subset of bovine antibodies with an extended heavy chain complementary-determining region loop (CDR) H3 (30). Crystal structure analysis has revealed that the ultra-long CDR H3 features unique “stalk-and-knob” architecture comprising a “stalk” made up of two antiparallel β-strands crowned by a globular disulphide-rich “knob” (**Figure 1A**) (31–35). Despite similar architectures, the sequences of ultra-long CDR H3 are astonishingly diverse (31). Such a diversity originates predominantly from somatic hypermutation and has potentially evolved as compensatory mechanism to counteract a comparative scarcity of bovine immune gene segments within the heavy chain repertoire (31, 36). Consistent with that, ultra-long CDR H3 loops play a pivotal role in antigen binding (31–37).

We have recently shown that the central segment of bovine ultra-long CDR H3 or “knob” (knob domain peptide) can independently bind to antigen with high affinity and specificity and therefore represents the smallest autonomous antibody fragment (3-6 kDa) (37). A stabilising network of disulphide bonds may confer on them high thermostability (37) and resistance to plasma proteolysis (38). Their extremely small size and the ability to exploit the bovine immune system for targeting a vast range of antigens make knob domain peptides a prospective new class of drugs that would address the limitations of mAbs rooted in their dimensions and structural complexity: restricted tissue penetration (39), aggravated adverse on/off-target effects due to slow elimination (40), immunogenicity (41) and high costs of manufacture (42).

The single-domain nature of knob domain peptides makes them particularly attractive for generation of bispecific constructs that would be naturally free from issues with the unbalanced expression of chains and promiscuous heavy-light chain paring. Unlike the other single-domain antibody fragments (VHH and VNAR) (26, 29), the knob domain paratope is not adjacent to N- or C-terminus (35, 43). This may enable construction of head-to-tail fusions free from the binding site steric occlusion. Moreover, the proximity of N- and C-termini makes knob domains well suited for grafting onto loops of various proteins, including conventional antibody fragments (44). Five different formats of bispecific knob domain fusions have been described to date: knob domains transplanted into a framework II loop of a VHH (44), framework III loop of a Fab fragment (45), and either AB or EF loops of a full-length IgG CH3 domain (46), as well as an IgG-like bispecific format described in (47). However, all these formats combine a knob domain with a larger antibody fragment and therefore do not leverage small size of the former in full.

A major challenge facing development of knob domain-only bispecifics is the lack of efficient production platform. Recombinant knob domain peptides express poorly both on their own and as entire CDR H3 comprising the “knob” and the “stalk” (37). Expression yields can be rescued by tethering to a larger protein, such as human Fc or thioredoxin (37, 43, 48), or by inserting into a loop as described above (37, 44–46). Upon separation from fusion partners, the knob domain peptides retain their antigen-binding activity (37, 43). Another approach, described in (38), utilises solid-phase peptide synthesis (SPPS) to produce individual knob domain peptides devoid of stalk and any additional fusion tags. Compared to recombinant protein technology, SPPS provides an easy way to incorporate some therapeutically relevant modifications, such as noncanonical amino acids, palmitoylation and head-to-tail cyclisation. However, both the approaches are associated with higher costs when scaling up production levels. The manufacture of knob domains as fusions to large proteins requires additional steps for tag removal and purification of cleavage products hindering commercial viability of the product (49). The cost of chemical synthesis can be equally prohibitive in the case of long peptides or when large quantities are required (50, 51).

In this paper, we present a modular platform for engineering of bi- and multispecific knob domain-only fusions based on the substitution of a natural β-ribbon stalk with antiparallel coiled-coil dimers. We demonstrate that the resulting constructs can be recombinantly expressed at high yield in mammalian cells and do not require any additional purification steps that would hamper large-scale production. The constructs are capable of simultaneous binding to all their target antigens and retain high binding affinities exhibited by individual knob domains.

## 2 Materials and methods

### 2.1 Knob domain discovery

The knob domain peptide aIL2_1 targeting IL-2 protein was discovered through phage display following the procedure described in (52). Briefly, immune cells isolated from a lymph node of an adult Friesian cow immunised with human IL-2 were used for total RNA extraction and subsequent amplification of knob domain-coding sequences with a mixture of stalk-specific primers. The PCR product was cloned into a phagemid vector as an N-terminal fusion to the pIII coat protein. After two rounds of panning on 100 nM IL-2, phage clones were screened by ELISA identifying ten unique IL-2-specific knob domain sequences. The knob domain peptide derived from the most enriched sequence (aIL2_1, TTVHQSTRTRESCPESYRFHSDRWSRNCCIPDSWDDSYVWNCDHYAVRPAISAYTYENHVDA) was synthetised by solid-phase peptide synthesis, and its binding to IL-2 was confirmed in an SPR experiment.

The discovery of anti-complement component 5 (C5) knob domain peptides has been previously reported (37).

### 2.2 Vectors

The knob domains mammalian expression constructs used in this study were N-terminally fused with a leader peptide derived from murine immunoglobulin heavy chain (MEWSWVFLFFLSVTTGVHS, UniProt A0N1R4_MOUSE) and C-terminally fused with an 8×His tag. The constructs for bacterial expression were supplied with a C-terminal 8×His tag only. DNA sequences were codon-optimised for the respective species, synthetised and cloned into either pcDNA3.1(+) (HindIII-XhoI) or pET-26b(+) (NdeI-XhoI) vector by GenScript.

### 2.3 Screening of knob domain expression constructs

Expression screening was conducted by transiently transfecting Expi293F cells (Gibco) using ExpiFectamine 293 Transfection Kit (Gibco), as per manufacturer’s instructions. Briefly, the cells were seeded in 24-well culture blocks as individual 2 mL cultures at 3 × 10^6^ cells/mL and transfected with 2 µg of plasmids. 96 h post-transfection, cell media were harvested by centrifugation at 16,000 g for 5 min. Secreted His-tagged knob domain constructs were enriched in an automated 12-channel PhyNexus MEA 2 protein purification system using PhyTip columns packed with 10 µl of Ni-IMAC resin (part number PTR-91-10-03). First, the columns were equilibrated with 1 mL of PhyNexus Capture Buffer (part number BUF-91-40-03). 900 µL of media samples were then mixed with 100 µL of 0.5 M sodium phosphate, 1.5 M NaCl, 100 mM imidazole (pH 8.0) and loaded on the columns by slowly pipetting up and down. The columns were washed two times with 1 mL of 1:4 diluted PhyNexus Wash Buffer, and bound fractions were eluted with 30 µL of PhyNexus Elution Buffer. The eluates were assayed for knob domain constructs by SDS-PAGE under reducing and non-reducing conditions.

### 2.4 Protein expression and purification in mammalian cells

Scale-up to 50 mL cultures was carried out in 250 mL Erlenmeyer flasks. To enable large-scale transfection, plasmid DNA was amplified using QIAGEN Plasmid Plus Maxi Kit. Culture media were harvested by centrifugation at 7,000 g for 1 h and passed through 0.22 µm filters. The knob domain constructs were purified by Ni-IMAC using an ÄKTA pure chromatography system (Cytiva) as previously described (37). Briefly, a 1 ml HisTrap excel column (Cytiva) was equilibrated with PBS, 0.5 M NaCl prior to loading cell supernatants. The column was extensively washed with the same buffer followed by another wash with PBS, 0.5 M NaCl, 20 mM imidazole. Bound proteins were eluted with PBS, 0.5 M NaCl, 200 mM imidazole. The protein-containing fractions were pooled, quantified by measuring absorbance at 280 nm and stored frozen at −80°C for subsequent analyses.

### 2.5 Protein expression and purification in bacterial cells

Plasmids encoding BECN1-fused knob domains were transformed into chemically competent SHuffle T7 *E. coli* cells (New England Biolabs) and plated onto LB-agar plates supplied with 50 µg/ml of kanamycin. The colonies were transferred into 2xTY, 50 µg/ml kanamycin and incubated at 37°C for 16 h. The resulting cultures were used to inoculate 3 L of fresh medium. Recombinant expression was induced by adding 1 mM IPTG at OD_600_ ~ 0.6, and the temperature was decreased to 18°C. After 16 h, the cells were harvested by centrifugation (7,000 g for 1 h), resuspended in PBS, 0.5 M NaCl, 4 M guanidinium chloride and lysed by passing through a French press. The lysate was cleared by centrifugation (20,000 g for 1 h), passed through 0.22 µm filters and loaded onto a HisTrap HP column (Cytiva) equilibrated with the lysis buffer. Bound proteins were refolded by running a linear 4-0 M guanidinium chloride gradient. After a wash with PBS, 0.5 M NaCl, 20 mM imidazole, the knob domains were eluted by PBS, 0.5 M NaCl, 200 mM imidazole. Pooled fractions containing the protein were purified by reversed-phase (RP) HPLC as described in (38). The product was lyophilised and stored at −20°C for subsequent analyses.

### 2.6 Solid-phase peptide synthesis

Knob domain peptides devoid of native stalk were synthesised using solid-phase peptide synthesis employing 9-fluorenylmethoxycarbonyl (Fmoc) as the α-amino protecting group. Disulphide bonds were formed by thermodynamically controlled air oxidation (38).

### 2.7 Concentration measurements

Purified proteins were quantified using a Trinean DropSense96 droplet reader taking absorbance measurements at 280 nm wavelength. The molar extinction coefficients of knob domain constructs were calculated from numbers of tryptophan, tyrosine and cystine residues using the molar extinction coefficients of individual amino acids determined by Edelhoch (53).

### 2.8 LC-MS

LC-MS analysis was performed using a Waters ACQUITY UPLC System connected to a Waters Xevo G2 Q-ToF mass spectrometer operated by MassLynx™ Software. Ni-IMAC eluate samples (5 µL at 0.05-0.4 mg/mL) were injected on a BioResolveT RP mAb Polyphenyl, 450 Å, 2.7 µm column held at 80°C with a flow rate of 0.6 mL/min. The mobile phase buffers were water, 0.02% trifluoroacetic acid (TFA), 0.08% formic acid (Solvent A) and 95% acetonitrile, 5% water, 0.02% TFA, 0.08% formic acid (solvent B). A reverse phase gradient was run from 5% to 50% solvent B over 8.8 min with a 95% solvent B wash and re-equilibration at 5% solvent B. UV data were acquired at 260-300 nm. For mass spectrometry, the system was configured as follows: ion mode, ESI positive; acquisition mode, resolution; mass range, 400–5,000 m/z; cone voltage, 30 V; capillary voltage, 3.2 kV; desolvation temperature, 350°C; desolvation gas, 1,000 L/h; source temperature, 150°C. Data analysis was performed using MassLynx™ and OpenLynx™ software.

### 2.9 HPLC

SEC-HPLC analysis was performed using Agilent 1100 Series HPLC system. Ni-IMAC eluate samples (20 µL at 0.05-0.4 mg/mL) were injected on a Tosoh Bioscience TSKgel G3000SWXL column (7.8 × 300 mm) running at 1 mL/min in 0.2 M sodium phosphate buffer, pH 7. Chromatograms were obtained by monitoring absorbance at 280 nm and fluorescence intensity at Ex/Em = 280/345 nm. Molecular weights were determined using a set of five protein standards (670 kDa, 158 kDa, 44 kDa, 17 kDa, 1.35 kDa).

### 2.10 ELISA

The antigen binding ability of knob domain constructs was tested by indirect sandwich ELISA. 96-well Nunc MaxiSorp plates were coated with 1-3 µg/mL solutions of either IL-2 (kindly provided by Louise Speight) or C5 (purified from serum as described in (54)) in PBS. The plates were blocked with 10% Aquatic Block Reagent (Millipore) in PBS and incubated with the dilutions of purified knob domain constructs in PBS, 10% Aquatic Block Reagent, 0.05% Tween-20. Detection was performed using 1:2,000 rabbit anti-6-His-Tag primary antibodies (Bethyl Laboratories) and 1:5,000 HRP-conjugated goat anti-rabbit secondary antibodies (Jackson ImmunoResearch). The washing steps comprised five wash cycles with PBS, 0.05% Tween-20. To reveal, the plates were incubated with 1-Step Ultra TMB-ELISA Substrate Solution (Thermo Scientific) and the optical density at 652 nm was measured using a BioTek Synergy Neo2 microplate reader.

### 2.11 SPR multicycle kinetics

The binding kinetics were measured using a Biacore 8K+ instrument. The antigens and knob domain constructs were immobilised on a Biacore CM5 chip via amine coupling; serial dilutions of their respective binding partners were prepared in HBS-EP+ buffer (10 mM HEPES pH 7.4, 150 mM NaCl, 3 mM EDTA, 0.005% v/v Surfactant P20). For each injection, a flow rate of 40 µL/min was used. Association was recorded for 300 s (K8 and K57) or 100 s (aIL2_1); dissociation was recorded for 6000 s (K8 and K57) or 1000 s (aIL2_1). The surface was regenerated by two sequential 30 s pulses of 2 M MgCl_2_. To determine the binding kinetics, the data obtained after subtraction of reference measurements were fitted to 1:1 (K8 and K57) or a two-state (aIL2_1) binding model using Biacore evaluation software. In the latter case, we report the fastest association rate and the slowest dissociation rate constants only.

## 3 Results

### 3.1 Construct design

Our previous experiments have shown that knob domains in isolation are poorly expressed in mammalian cells and that incorporation of either entire ultra-long CDR H3 region (Kabat H93-H102) comprising both knob domain and the stalk into single-chain human Fc or knob domain alone into CDR H3 region of human Fab can dramatically improve expression yields (37). We hypothesised that the proximity of N- and C-termini constrained by the stalk or immunoglobulin scaffold, respectively, facilitates the folding of knob domains, and an attempt to express a knob domain alone results in accumulation of misfolded protein aggregates not detectable in cell supernatant. Based on this assumption, we designed 23 minimalistic frameworks that were anticipated to enhance the expression of knob domains by constraining their termini in close proximity, including: knob domains with their native stalks, either full-length (8-10 aa) or shortened (4-6 aa), knob domains fused with the stalk from ultra-long CDR H3 Bov-5 (PDB 6E9K) (32) that has the highest number of interstrand side chain-side chain interactions among all ultra-long CDR H3 regions with known structure, knob domains with S-S-linked termini, knob domains with stalks stabilised by cross-strand disulphide bonds (55), knob domains grafted onto S-S-stabilised helix-helix motif from human serum albumin (HSA, residues 90-101, PDB 1BM0) and knob domains grafted onto stretches of antiparallel coiled-coil dimers taken from either *Sin Nombre orthohantavirus* nucleocapsid protein (SNV-N, residues 4-11 and 61-68, PDB 2IC6) or human proteins.

As an orthogonal approach, we explored whether attachment of solvent-exposed N- and C-terminal motifs from highly abundant proteins to knob domains can improve their expression levels by increasing the solubility. For that, we designed a series of 11 constructs comprising knob domains fused with the peptides taken from mature sequences of human immunoglobulin κ light chain (residues 1-3 or 1-7, UniProt P0DOX7), human immunoglobulin γ heavy chain (residues 1-4 or 1-8, UniProt P0DOX5), human haemoglobin beta subunit (HBB, residues 1-16, PDB 4N8T) and HSA (residues 1-13 or both 1-13 and 573-585, PDB 1BM0).

The ability of the synthetic frameworks to increase expression yields of knob domain peptides was evaluated by screening a panel of constructs comprising an anti-IL-2 knob domain peptide (aIL2_1) (52) and two unrelated anti-complement component 5 (C5) knob domain peptides (clones K8 and K57) (37) incorporated into each of the frameworks (**Supplementary Table S1**). Based on the available structures, we designated the residue immediately preceding the first germline cysteine as the N-terminal residue of the knob domain. In case of K57, we chose the second residue before the first cysteine as the ascending strand of the stalk (starting from H93) would otherwise comprise an unusual even number of amino acids. The C-terminal residues were identified assuming equal length of the ascending and descending strands of the stalk. All constructs were synthesised with a C-terminal 8×His purification tag.

### 3.2 Recombinant expression of knob domains is facilitated by substitution of stalk with antiparallel coiled-coil dimers

**Figure 2:**
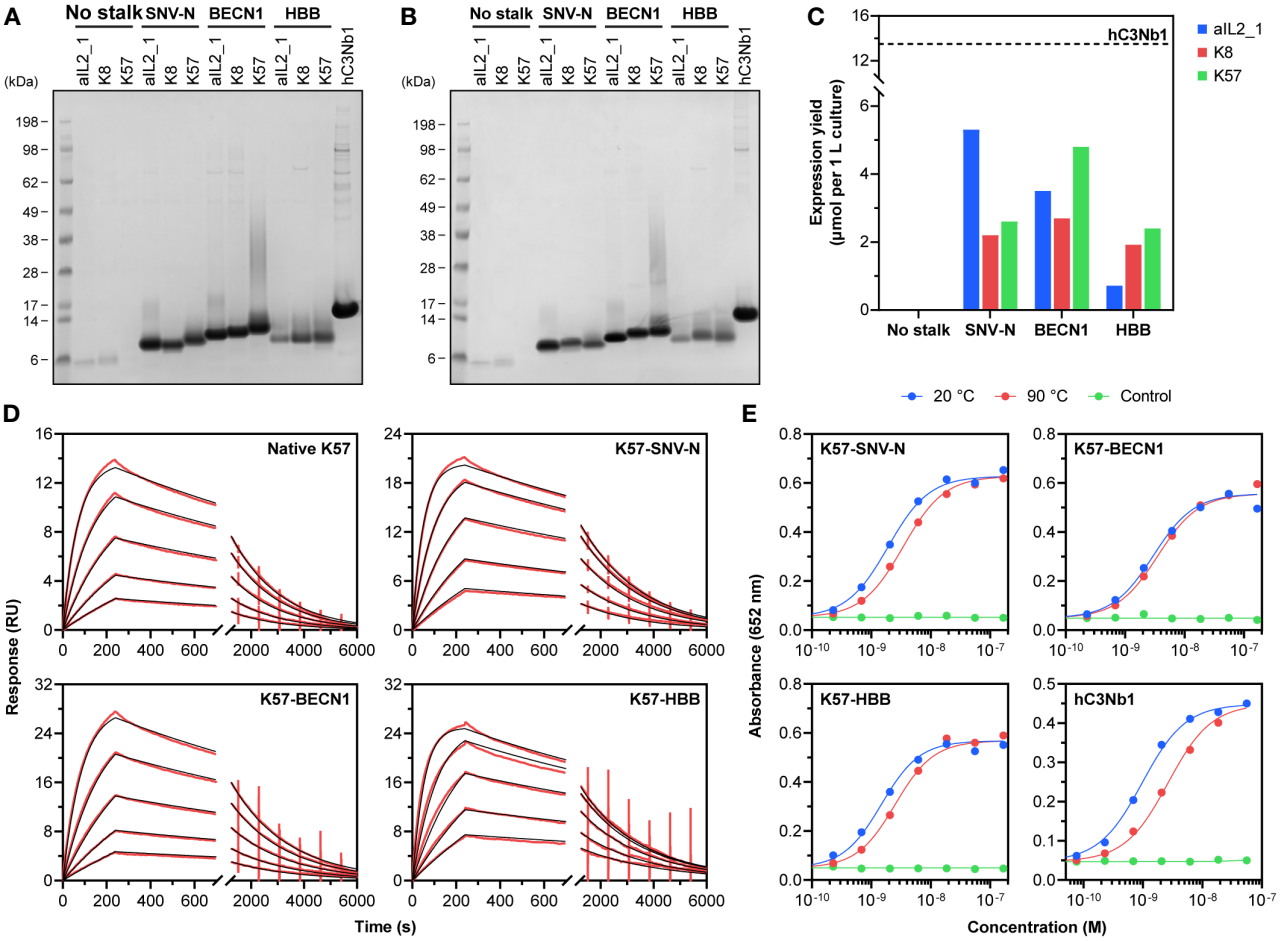
Purification and functional characterisation of recombinant knob domains. SDS-PAGE analysis of reduced **(A)** and unreduced **(B)** Ni-IMAC eluates confirms recombinant expression of the designed knob domain fusions; a representative VHH hC3Nb1 expressed and purified under similar conditions is shown for comparison. Theoretical molecular weights of SNV-N and HBB fusions are 7-8 kDa, theoretical molecular weights of BECN1 fusions are 10-11 kDa. **(C)** Molar expression yields of recombinant knob domains compared to the VHH. **(D)** Representative sensorgrams for binding of K57 alone and K57 fusion constructs to human C5. Immobilised C5 was subject to the injections of various concentrations of K57. Experimental curves (in red) were fitted with a 1:1 binding model (in black). **(E)** C5-binding activity of K57 fusion constructs and hC3Nb1 after a thermal stress. The proteins were kept at 20°C or heated to 90°C for 30 min and analysed by ELISA; uncoated blocked wells treated with 20°C samples served as negative control.

Expression screening was carried out as a series of small-scale transient transfections of Expi293F cells, to permit cell supernatants to be enriched by Ni-IMAC and analysed by SDS-PAGE. Plasmids encoding the knob domains devoid of stalks and knob domains with native stalks were used as negative controls. As expected, the control constructs expressed below detectable levels (**Figures 2A, B**; **Supplementary Figure S2**). Fusing the knob domains with antiparallel coiled-coiled dimers taken from SNV-N and human autophagy-related protein Beclin-1 (BECN1, residues 103-121 and 68-86, UniProt K7ELY9) dramatically improved expression yields of all three target knob domain peptides (**Figures 1B, C**, **2A, B**). Similar results were seen after N-terminal fusion with the first 16 residues taken from mature HBB sequence, as well as grafting both N- and C-terminal helices from HSA onto respective ends of the knob domains. All other frameworks displayed no or modest improvement for at least one out of three knob domain peptides.

We further assessed SNV-N, BECN1 and HBB constructs by scaling up the transfections to enable the accurate quantification of expression yield and biophysical characterisation of knob domain fusions. Purified constructs were tested to ascertain their identity and validate protein quality. SDS-PAGE analysis confirmed homogeneity of the samples, and we were unable to detect any misfolded disulphide cross-linked oligomers by repeating SDS-PAGE under non-reducing conditions (**Figure 2B**). Molecular weights determined by liquid chromatography-mass spectrometry (LC-MS) matched theoretical values calculated for monomeric proteins (**Supplementary Figure S3**). Interestingly, the mass spectra of HBB-fused knob domains exhibited peaks shifted by +948 Da from their respective theoretical values, consistent with the presence of common *O*-linked tetrasaccharide -GalNAc(-NeuNAc)-Gal-NeuNAc occurring in recombinant proteins produced by mammalian kidney cell lines (56, 57). Analysis by SEC-HPLC confirmed the monomer purity indicating that all samples were free from high molecular weight aggregates (**Supplementary Figure S4**).

Expression levels of knob domain constructs were benchmarked against a His-tagged version of well-characterised complement component 3-specific VHH hC3Nb1, which can be expressed in Expi293F cells at exceptionally high level (44, 58). We obtained 6-50 mg of purified knob domains per 1 L culture, compared to approximately 195 mg of hC3Nb1, but due to the smaller size of knob domains, hC3Nb1 has only a 2.5-fold molar advantage over the highest expressed knob domain aIL2_1-SNV-N (13.5 µmol per 1 L culture of hC3Nb1 compared to 5.3 µmol per 1 L culture of aIL2_1-SNV-N; **Figure 2C**).

### 3.3 Knob domains with artificial coiled-coil stalks exhibit high binding affinity to target antigens

We next asked whether recombinant knob domains expressed as SNV-N, BECN1 and HBB fusions retain their native-like conformation. To test this, we assayed the antigen-binding ability of the constructs using ELISA. All K8 and K57 constructs were found to bind to C5 and all aIL2_1 constructs displayed binding to IL-2 (**Supplementary Figure S5**). More detailed data on antigen binding kinetics were obtained via multicycle SPR experiments, by comparing recombinant knob domains fusions with knob domains alone produced by solid-phase peptide synthesis. Consistent with the published data (37, 38, 44), K8 and K57 exhibited tight binding to C5, with *K*_D_ values in low nanomolar range and relatively slow on- and off-rate kinetics (**Table 1**, **Figure 2D**; **Supplementary Figure S6**). In the specific case of K57, incorporation into the BECN1 framework led to a fourfold decrease in the binding affinity (mainly due to a slower association rate constant), and the HBB framework reduced the binding affinity of K8 by a factor of five (with contributions from both association and dissociation rate constants). With these exceptions, incorporation of knob domains in any of the expression frameworks had little or no effect on the binding. These data suggest that recombinant knob domain constructs retain their native structure, in a manner consistent with antigen binding.

**Table 1:** Antigen-binding kinetics of knob domains produced by solid-phase peptide synthesis (aIL2_1, K8 and K57) and recombinant knob domain fusion constructs.

Construct	*k*_a_, M^-1^s^-1^	*k*_d_, s^-1^	*K*_D_, M
aIL2_1	6.0 × 10^5^	3.2 × 10^-3^	9.0 × 10^-9^
aIL2_1-SNV-N	2.3 × 10^5^	2.5 × 10^-3^	1.79 × 10^-8^
aIL2_1-BECN1	1.43 × 10^5^	1.85 × 10^-3^	2.3 × 10^-8^
aIL2_1-HBB	4.7 × 10^5^	3.1 × 10^-3^	1.01 × 10^-8^
K8	6.2 × 10^4^	2.3 × 10^-4^	3.8 × 10^-9^
K8-SNV-N	9.8 × 10^4^	4.7 × 10^-4^	4.8 × 10^-9^
K8-BECN1	3.8 × 10^4^	2.8 × 10^-4^	7.5 × 10^-9^
K8-HBB	2.6 × 10^4^	5.1 × 10^-4^	1.95 × 10^-8^
K57	2.3 × 10^5^	5.4 × 10^-4^	2.4 × 10^-9^
K57-SNV-N	1.55 × 10^5^	4.5 × 10^-4^	2.9 × 10^-9^
K57-BECN1	5.2 × 10^4^	5.3 × 10^-4^	1.02 × 10^-8^
K57-HBB	9.4 × 10^4^	5.3 × 10^-4^	5.6 × 10^-9^

### 3.4 Knob domains with artificial coiled-coil stalks demonstrate high thermal stability

To evaluate the ability of the knob domain constructs to maintain their performance upon thermal stress, we analysed their antigen-binding ability after a 30 min exposure to 90°C. The samples were diluted to 5 µM in PBS and subjected to heating. Cooled samples were briefly centrifuged (16,000 g, 5 min) to remove protein aggregates, and the supernatants were assessed by ELISA. All knob domain constructs displayed high levels of thermal stability: the binding activity either remained constant (K8-BECN1) or moderately declined (**Figure 2E**; **Supplementary Figure S7**). This compares favourably to hC3Nb1, which suffered a threefold decline in antigen binding (**Figure 2E**).

### 3.5 Knob domains with BECN1 stalk can be recombinantly produced in bacterial cells

Next, we set out to investigate whether the knob domains with artificial coiled-coil stalks can be recombinantly produced in procaryotic systems. We employed SHuffle T7 *E. coli* cells (New England Biolabs) designed to enhance the capacity for folding of disulphide-rich proteins. This enabled us to express BECN1 fusions of aIL2_1, K8 and K57 in bacterial cytoplasm that were subsequently isolated using a combination of Ni-IMAC and RP-HPLC. Purified constructs showed up as monomers on a non-reducing SDS-PAGE gel (**Supplementary Figure S8**) and exhibited high antigen-binding affinities similar to knob domains manufactured in mammalian cells. However, expression yields were relatively low ranging from 4 to 12 mg per 1 g of wet cell mass.

### 3.6 Coiled-coil dimers enable construction of bispecific knob domain fusions

**Figure 3:**
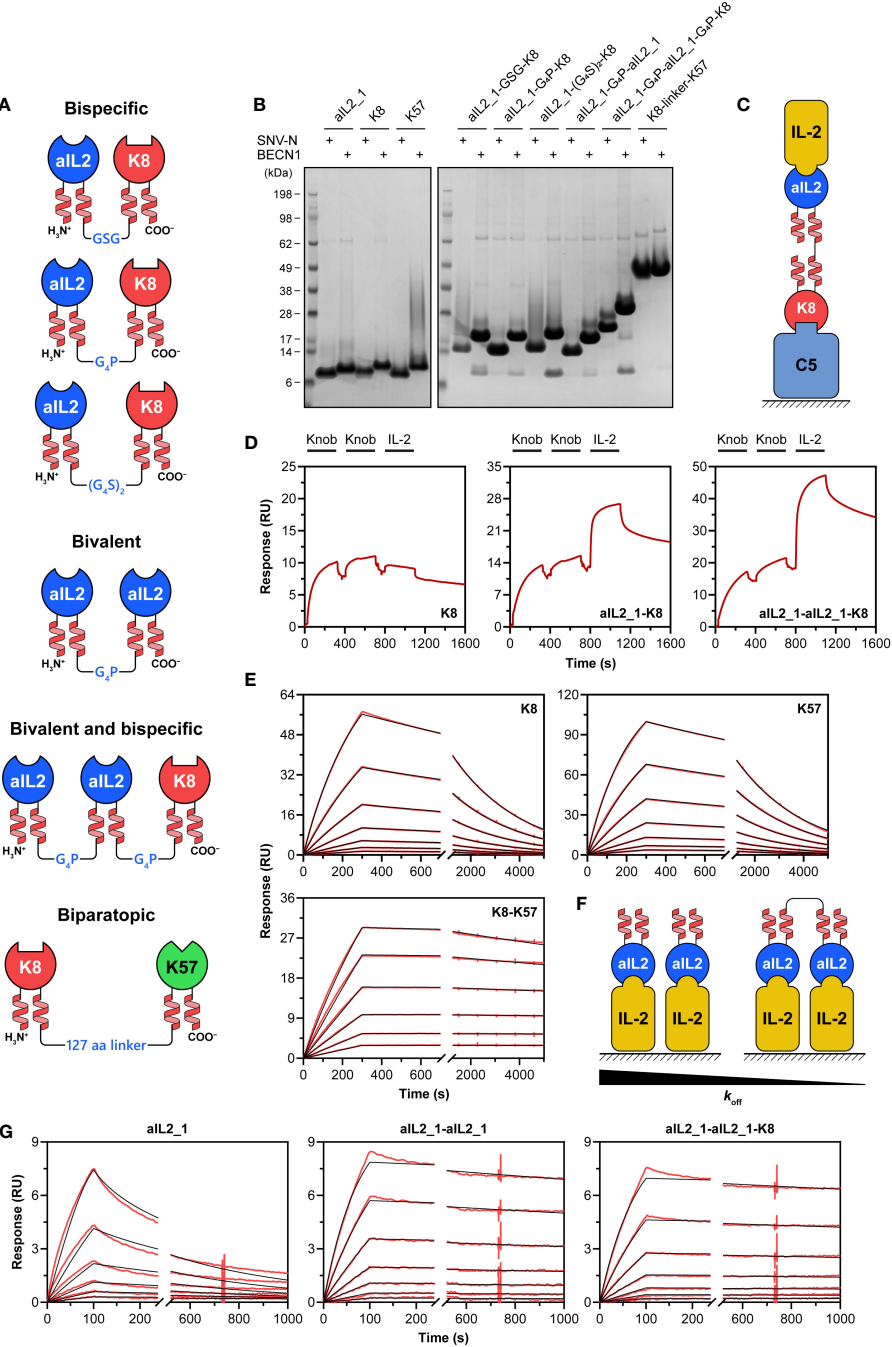
Bispecific knob domain fusions. **(A)** Schematic representation of the designed bispecific, bivalent and biparatopic knob domain fusions. **(B)** SDS-PAGE analysis of knob domain fusions expressed in Expi293F cells. The knob domains were supplied with either SNV-N or BECN1 stalks; individual knob domains were expressed as controls. The gels were run under non-reducing conditions. Migration of K8-K57 fusions (SNV-N, 24 kDa; BECN1, 30 kDa) is slowed down by an extensive glycine-rich linker. **(C)** Schematic representation of bridging SPR assay used in this study. The surface is coated with one of the antigens (C5), followed by the addition of bispecific fusion. The second antigen (IL-2) is then added. Dissociation of the complex is measured by washing the surface with buffer. **(D)** The bridging SPR assay confirms simultaneous binding of SNVN-fused bispecific knob domain constructs to their antigens. **(E)** Representative sensorgrams for binding of SNV-N-fused anti-C5 biparatopic K8-K57 knob domain construct to C5. Individual K8 and K57 were used as controls. Immobilised knob domains were subject to the injections of various concentrations of C5. Experimental curves are shown in red, and the fitted curves are shown in black. **(F)** Schematic representation of IL-2 binding assay used to confirm simultaneous engagement of two target molecules by bivalent fusions. The surface is coated with an antigen (IL-2), followed by the addition of knob domain constructs. An increase in valency would be associated with a decrease in dissociation rate (koff). **(G)** Representative sensorgrams for binding of SNV-N-fused bivalent anti-IL-2 constructs to IL-2. Individual IL-2 was used as control. Immobilised IL-2 was subject to the injections of various concentrations of knob domain constructs. Experimental curves are shown in red, and the fitted curves are shown in black.

After demonstrating that functional knob domains with artificial coiled-coil stalks can be efficiently produced in recombinant systems, we wished to explore whether they can be used as modular building blocks for generation of bispecific fusions. As a proof-of-concept, we designed a panel of constructs based on the SNV-N- and BECN1-fused knob domains (**Figure 3A**), including: bispecific aIL2_1-K8 fusions joined via flexible linkers of varying length (GSG, GGGGP and GGGGSGGGGS), bivalent aIL2_1-aIL2_1 fusions joined via GGGGP linker, bispecific/bivalent aIL2_1-aIL2_1-K8 fusions joined via GGGGP linkers and biparatopic K8-K57 fusions joined via 127 amino acid-long linker comprising a tandem repeat of a sequence that we have previously developed as part of a single-chain Fc polypeptide (59). Such an extensive linker was required to cover a 100 Å distance between K8 and K57 epitopes located on the MG8 and MG5 domains of the C5 protein (PDB 3CU7) (35).

All the designed constructs expressed at high levels in Expi293F cells and appeared as well-folded monomers on SDS-PAGE gel under non-reducing conditions (**Figure 3B**). Molecular weights determined by LC-MS confirmed the identity and the monomeric state of the samples (**Supplementary Figure S9**).

Next, we aimed at evaluating the antigen-binding activity. All constructs were able to engage their targets in an ELISA assay (**Supplementary Figure S10**). The binding kinetics of aIL2_1-K8 fusions determined by SPR were nearly identical to the parental knob domains (**Supplementary Table S11**, **Supplementary Figure S12**). Having confirmed individual binding to IL-2 and C5, we set out to investigate whether aIL2_1-K8 and aIL2_1-aIL2_1-K8 could bind to IL-2 and C5 simultaneously. For that, we employed a bridging SPR assay described in (60). Briefly, immobilised C5 was subjected to binding cycles of the knob domain fusions followed by a binding cycle of IL-2 (**Figure 3C**). A single knob domain K8 was used as a control. While all samples showed initial association with C5, only aIL2_1-K8 and aIL2_1-aIL2_1-K8 were able to subsequently associate with IL-2 (**Figure 3D**; **Supplementary Figure S13**). It is worth noting that the increase in signal upon IL-2 binding by aIL2_1-aIL2_1-K8 is roughly double compared to aIL2_1-K8 (26 vs. 12 RU) indicating formation of quaternary complex.

To demonstrate simultaneous binding of biparatopic K8-K57 fusions to MG8 and MG5 domains of C5, we compared its binding kinetics to the kinetics of individual knob domains. We anticipated the fusions to form intra- and intermolecular bridges that would enhance the binding affinity (61, 62). Indeed, the dissociation of K8-K57 from C5 was about an order of magnitude slower than the most stable individual knob domain (**Figure 3E**; **Supplementary Table S11**; **Supplementary Figure S14**). The observed association rates of biparatopic fusions were not compromised compared to K8 and K57.

Similarly, fusing two aIL2_1 knob domains into bivalent constructs led to substantial slowing down of dissociation from IL-2-coated surface (up to two orders of magnitude, **Figures 3F, G**; **Supplementary Table S11**; **Supplementary Figure S15**). This is due to the fusions being able to simultaneously engage two IL-2 molecules on the solid phase leading to enhanced binding avidity. As expected, dissociation rates of the bivalent constructs were relatively close to dissociation rate of individual aIL2_1 when the antigen was flowed over the knob domain-coated chip surface (**Supplementary Table S11**, **Supplementary Figure S12B**).

## 4 Discussion

Here, we have shown that recombinant knob domains can be efficiently produced in mammalian and bacterial cells as fusions to short helical peptides acting as alternative stalks. From over thirty potential constructs, we have developed a total of three expression frameworks, each compatible with all three tested knob domain peptides. Following transient transfection, the designed frameworks succeeded in yielding tens of mg of protein per litre of culture, comparable to the expression level of a selected VHH fragment hC3Nb1. These numbers are also in line with previously reported yields of recombinant antibodies and antibody fragments in other transient Expi293F expression systems (63–67). Due to their small size, knob domains expressed in this manner offer a molar yield that is comparable to conventional antibodies.

Maintaining the native structure of recombinant proteins is one of the key factors affecting expression levels (68). To date, a coiled-coil module has only been used to substitute for a knob domain stalk in the context of insertion of granulocyte colony-stimulating factor peptide into CDR H3 of full-length bovine IgG (69). In contrast, with this paper we show that coiled-coil dimers enable the expression of autonomous knob domain peptides in the absence of acceptor protein infrastructure.

Another approach that was examined in this study is incorporation of N-terminal sequences of naturally highly expressed proteins into the N-terminus of knob domains. Similar strategies have been previously used for high-yield production of recombinant proteins in microbial hosts (70–73), however, to our knowledge, there are no reports on using such solubility tags for enhancing recombinant protein expression in mammalian cells. We have shown that expression of knob domains can be rescued by tethering to a 15 amino acid-long N-terminal α-helix from human haemoglobin subunit beta. The increase in recombinant protein yield may be attributed to *O*-linked glycosylation of haemoglobin helix that can enhance the solubility of fusion constructs and prevent them from aggregation (74).

The knob domain expression strategies proposed in this study employ relatively short peptide tags rather than large protein fusions such as a C-terminal fusion to the IgG Fc fragment that we previously described (37). Using short tags not only reduces metabolic burden on the host cells, but, crucially, allows commercial-scale production of knob domains by removing the extra costs associated with cleavage of large fusions that can disrupt therapeutic function and subsequent purification steps (49). Small size of our peptide tags would also prevent them from altering pharmacokinetic characteristics of knob domains. As evidenced by several independent techniques, the designed frameworks do not compromise the antigen-binding capacity. On top of that, all the constructs described displayed impressive heat tolerance, functioning after prolonged exposure to 90°C. Such stability may ultimately reduce requirements for stringent cold chain storage and distribution for products featuring knob domains with artificial coiled-coil stalks.

We have recently demonstrated that knob domain peptides can be grafted onto VHH and Fab fragments to generate functional fusions (44, 45), and the group led by Krah has successfully engineered bispecific bivalent fusions of knob domains to the full-length IgG (46). Here, we show that the knob domains with artificial coiled-coil stalks can serve as modular building units for knob domain-knob domain bispecifics. The molecular mass of the smallest fusion that we obtained is 14.5 kDa, which is about the mass of a single VHH fragment and four times as small as the most compact bispecific platform based on conventional antibody fragments – a diabody (23). Due to their size, the knob domain fusions may offer superior tissue penetration properties and improved access to cryptic epitopes (75). Intrinsic biophysical properties make knob domains especially well-suited to local delivery in potent combinations, where their short circulating half-lives may be expected to preclude systemic toxicity. On the other hand, the knob domain fusions containing an anti-albumin component (45) will have extended serum half-lives while retaining advantages of the small size. Importantly, such fusions can be readily made in recombinant systems and, being single-chain peptides, will avoid the issues with unbalanced expression levels of chains and their incorrect assembly.

Taken together, our platform enables efficient engineering of modular bi- and multi-specific knob domain-only fusions. The designed artificial stalks can also be used to achieve high-yield large-scale production of individual knob domains. In addition to applications as protein therapeutics, multi-knob fusions are likely to find utility as efficient gene therapy payloads.

## Data availability statement

The original contributions presented in the study are included in the article/**Supplementary Material**. Further inquiries can be directed to the corresponding author.

## Ethics statement

The animal study was approved by University of Reading Animal Welfare Ethical Review Body. The study was conducted in accordance with the local legislation and institutional requirements.

## Author contributions

MK: Conceptualization, Formal analysis, Investigation, Methodology, Writing – original draft, Writing – review & editing. GG: Investigation, Methodology, Writing – original draft. CJ: Formal analysis, Investigation, Methodology, Writing – review & editing. AS-T: Conceptualization, Methodology, Supervision, Writing – review & editing. AM: Conceptualization, Methodology, Supervision, Writing – original draft, Writing – review & editing. AL: Conceptualization, Methodology, Supervision, Writing – original draft, Writing – review & editing.
