# Supplementary material for: Modular design of bi- and multi-specific knob domain fusions
Source: Front Immunol. 2024 Mar 27;15:1384467. doi: 10.3389/fimmu.2024.1384467 (PMC11008599; doi:10.3389/fimmu.2024.1384467)
Supplement: Supplementary file 1 [file DataSheet_1.docx]

Supplementary Material

**Supplementary Table S1.** Amino acid sequences of the screened knob domains expression constructs.

| Knob domain | Framework | Sequence |
| --- | --- | --- |
| aIL2_1 | Full-length native stalk | TTVHQSTRTRESCPESYRFHSDRWSRNCCIPDSWDDSYVWNCDHYAVRPAISAYTYENHVDHHHHHHHH |
| K8 |  | QQKTHQVCPDGFNWGYGCAAGSSRFCTRHDWCCYDERADSHTYGFCTGNRVTNTYEFHADHHHHHHHH |
| K57 |  | TVHQRTIKSGCPPGYKSGVDCSPGSECKWGCYAVDGRRYGGYGADSGVGSTYTHEFYVDHHHHHHHH |
| aIL2_1 | Shortened native stalk | STRTRESCPESYRFHSDRWSRNCCIPDSWDDSYVWNCDHYAVRPAISAYTYEHHHHHHHH |
| K8 |  | KTHQVCPDGFNWGYGCAAGSSRFCTRHDWCCYDERADSHTYGFCTGNRVTNTYEHHHHHHHH |
| K57 |  | RTIKSGCPPGYKSGVDCSPGSECKWGCYAVDGRRYGGYGADSGVGSTYTHEHHHHHHHH |
| aIL2_1 | S-S-cyclised shortened native stalk | SCRTRESCPESYRFHSDRWSRNCCIPDSWDDSYVWNCDHYAVRPAISAYTCEHHHHHHHH |
| K8 |  | KCHQVCPDGFNWGYGCAAGSSRFCTRHDWCCYDERADSHTYGFCTGNRVTNTCEHHHHHHHH |
| K57 |  | RCIKSGCPPGYKSGVDCSPGSECKWGCYAVDGRRYGGYGADSGVGSTYTCEHHHHHHHH |
| aIL2_1 | Knob domain only | SCPESYRFHSDRWSRNCCIPDSWDDSYVWNCDHYAVRPAIHHHHHHHH |
| K8 |  | VCPDGFNWGYGCAAGSSRFCTRHDWCCYDERADSHTYGFCTGNRVTHHHHHHHH |
| K57 |  | SGCPPGYKSGVDCSPGSECKWGCYAVDGRRYGGYGADSGVGSTHHHHHHHH |
| aIL2_1 | Full-length native stalk from BOV5 (C149S); PDB 6E9K, residues 97-106 & 141-150 | TVHQKTRKTFSCPESYRFHSDRWSRNCCIPDSWDDSYVWNCDHYAVRPAILTDNYEWYSDHHHHHHHH |
| K8 |  | TVHQKTRKTFVCPDGFNWGYGCAAGSSRFCTRHDWCCYDERADSHTYGFCTGNRVTLTDNYEWYSDHHHHHHHH |
| K57 |  | TVHQKTRKTFSGCPPGYKSGVDCSPGSECKWGCYAVDGRRYGGYGADSGVGSTLTDNYEWYSDHHHHHHHH |
| aIL2_1 | S-S-cyclised stalk from BOV5 (V98C); PDB 6E9K, residues 98-106 & 141-149 | CHQKTRKTFSCPESYRFHSDRWSRNCCIPDSWDDSYVWNCDHYAVRPAILTDNYEWYCHHHHHHHH |
| K8 |  | CHQKTRKTFVCPDGFNWGYGCAAGSSRFCTRHDWCCYDERADSHTYGFCTGNRVTLTDNYEWYCHHHHHHHH |
| K57 |  | CHQKTRKTFSGCPPGYKSGVDCSPGSECKWGCYAVDGRRYGGYGADSGVGSTLTDNYEWYCHHHHHHHH |
| aIL2_1 | Shortened stalk from BOV5; PDB 6E9K, residues 101-106 & 141-146 | KTRKTFSCPESYRFHSDRWSRNCCIPDSWDDSYVWNCDHYAVRPAILTDNYEHHHHHHHH |
| K8 |  | KTRKTFVCPDGFNWGYGCAAGSSRFCTRHDWCCYDERADSHTYGFCTGNRVTLTDNYEHHHHHHHH |
| K57 |  | KTRKTFSGCPPGYKSGVDCSPGSECKWGCYAVDGRRYGGYGADSGVGSTLTDNYEHHHHHHHH |
| aIL2_1 | S-S-cyclised | CGSSCPESYRFHSDRWSRNCCIPDSWDDSYVWNCDHYAVRPAIGSCHHHHHHHH |
| K8 |  | CGSVCPDGFNWGYGCAAGSSRFCTRHDWCCYDERADSHTYGFCTGNRVTGSCHHHHHHHH |
| K57 |  | CGSSGCPPGYKSGVDCSPGSECKWGCYAVDGRRYGGYGADSGVGSTGSCHHHHHHHH |
| aIL2_1 | S-S-cyclised helix-helix motif from human serum albumin (C115S); UniProt P02768, residues 114-118 & 119-125 | CSAKQGSSCPESYRFHSDRWSRNCCIPDSWDDSYVWNCDHYAVRPAIGSEPERNECHHHHHHHH |
| K8 |  | CSAKQGSVCPDGFNWGYGCAAGSSRFCTRHDWCCYDERADSHTYGFCTGNRVTGSEPERNECHHHHHHHH |
| K57 |  | CSAKQGSSGCPPGYKSGVDCSPGSECKWGCYAVDGRRYGGYGADSGVGSTGSEPERNECHHHHHHHH |
| aIL2_1 | Anti-parallel coiled-coil stalk from *Sin Nombre orthohantavirus* nucleocapsid protein; PDB 2IC6, residues 4-11 & 61-68 | LKEVQDNIGSSCPESYRFHSDRWSRNCCIPDSWDDSYVWNCDHYAVRPAIGSLKRELADLHHHHHHHH |
| K8 |  | LKEVQDNIGSVCPDGFNWGYGCAAGSSRFCTRHDWCCYDERADSHTYGFCTGNRVTGSLKRELADLHHHHHHHH |
| K57 |  | LKEVQDNIGSSGCPPGYKSGVDCSPGSECKWGCYAVDGRRYGGYGADSGVGSTGSLKRELADLHHHHHHHH |
| aIL2_1 | Anti-parallel coiled-coil stalk from human IL-2; PDB 2B5I, residues 18-28 & 115-122 | LLDLQMILNGIGSSCPESYRFHSDRWSRNCCIPDSWDDSYVWNCDHYAVRPAIGSVEFLNRWIHHHHHHHH |
| K8 |  | LLDLQMILNGIGSVCPDGFNWGYGCAAGSSRFCTRHDWCCYDERADSHTYGFCTGNRVTGSVEFLNRWIHHHHHHHH |
| K57 |  | LLDLQMILNGIGSSGCPPGYKSGVDCSPGSECKWGCYAVDGRRYGGYGADSGVGSTGSVEFLNRWIHHHHHHHH |
| aIL2_1 | Anti-parallel coiled-coil stalk from human IL-6; PDB 2IL6, residues 30-37 & 168-179 | IRYILDGIGSSCPESYRFHSDRWSRNCCIPDSWDDSYVWNCDHYAVRPAIGSLRSFKEFLQSSLHHHHHHHH |
| K8 |  | IRYILDGIGSVCPDGFNWGYGCAAGSSRFCTRHDWCCYDERADSHTYGFCTGNRVTGSLRSFKEFLQSSLHHHHHHHH |
| K57 |  | IRYILDGIGSSGCPPGYKSGVDCSPGSECKWGCYAVDGRRYGGYGADSGVGSTGSLRSFKEFLQSSLHHHHHHHH |
| aIL2_1 | Anti-parallel coiled-coil stalk from human thrombopoietin; PDB 1V7N, residues 60-71 & 97-108 | AQDILGAVTLLLGSSCPESYRFHSDRWSRNCCIPDSWDDSYVWNCDHYAVRPAIGSVRLLLGALQSLLHHHHHHHH |
| K8 |  | AQDILGAVTLLLGSVCPDGFNWGYGCAAGSSRFCTRHDWCCYDERADSHTYGFCTGNRVTGSVRLLLGALQSLLHHHHHHHH |
| K57 |  | AQDILGAVTLLLGSSGCPPGYKSGVDCSPGSECKWGCYAVDGRRYGGYGADSGVGSTGSVRLLLGALQSLLHHHHHHHH |
| aIL2_1 | Anti-parallel coiled-coil stalk from human thrombopoietin; PDB 1V7N, residues 60-67 & 101-108 | AQDILGAVGSSCPESYRFHSDRWSRNCCIPDSWDDSYVWNCDHYAVRPAIGSLGALQSLLHHHHHHHH |
| K8 |  | AQDILGAVGSVCPDGFNWGYGCAAGSSRFCTRHDWCCYDERADSHTYGFCTGNRVTGSLGALQSLLHHHHHHHH |
| K57 |  | AQDILGAVGSSGCPPGYKSGVDCSPGSECKWGCYAVDGRRYGGYGADSGVGSTGSLGALQSLLHHHHHHHH |
| aIL2_1 | Anti-parallel coiled-coil stalk from human spartin; PDB 1V7N, residues 45-52 & 84-91 | YKQGIGHLGSSCPESYRFHSDRWSRNCCIPDSWDDSYVWNCDHYAVRPAIGSLQNVRTRLHHHHHHHH |
| K8 |  | YKQGIGHLGSVCPDGFNWGYGCAAGSSRFCTRHDWCCYDERADSHTYGFCTGNRVTGSLQNVRTRLHHHHHHHH |
| K57 |  | YKQGIGHLGSSGCPPGYKSGVDCSPGSECKWGCYAVDGRRYGGYGADSGVGSTGSLQNVRTRLHHHHHHHH |
| aIL2_1 | Anti-parallel coiled-coil stalk from human ferritin heavy chain (C102S); PDB 4OYN, residues 97-107 & 145-156 | LNAMESALHLEGSSCPESYRFHSDRWSRNCCIPDSWDDSYVWNCDHYAVRPAIGSIKELGDHVTNLRHHHHHHHH |
| K8 |  | LNAMESALHLEGSVCPDGFNWGYGCAAGSSRFCTRHDWCCYDERADSHTYGFCTGNRVTGSIKELGDHVTNLRHHHHHHHH |
| K57 |  | LNAMESALHLEGSSGCPPGYKSGVDCSPGSECKWGCYAVDGRRYGGYGADSGVGSTGSIKELGDHVTNLRHHHHHHHH |
| aIL2_1 | Anti-parallel coiled-coil stalk from human ferritin heavy chain (C102S, E107I); PDB 4OYN, residues 97-107 & 145-156 | LNAMESALHLIGSSCPESYRFHSDRWSRNCCIPDSWDDSYVWNCDHYAVRPAIGSIKELGDHVTNLRHHHHHHHH |
| K8 |  | LNAMESALHLIGSVCPDGFNWGYGCAAGSSRFCTRHDWCCYDERADSHTYGFCTGNRVTGSIKELGDHVTNLRHHHHHHHH |
| K57 |  | LNAMESALHLIGSSGCPPGYKSGVDCSPGSECKWGCYAVDGRRYGGYGADSGVGSTGSIKELGDHVTNLRHHHHHHHH |
| aIL2_1 | Anti-parallel coiled-coil stalk from human Rabenosyn-5; PDB 1YZM, residues 459-473 & 482-496 | LLQQIHNITSFIRQAGSSCPESYRFHSDRWSRNCCIPDSWDDSYVWNCDHYAVRPAIGSVRTLQENLRQLQDEYHHHHHHHH |
| K8 |  | LLQQIHNITSFIRQAGSVCPDGFNWGYGCAAGSSRFCTRHDWCCYDERADSHTYGFCTGNRVTGSVRTLQENLRQLQDEYHHHHHHHH |
| K57 |  | LLQQIHNITSFIRQAGSSGCPPGYKSGVDCSPGSECKWGCYAVDGRRYGGYGADSGVGSTGSVRTLQENLRQLQDEYHHHHHHHH |
| aIL2_1 | Anti-parallel coiled-coil stalk from human Rabenosyn-5; PDB 1YZM, residues 463-470 & 485-492 | IHNITSFIGSSCPESYRFHSDRWSRNCCIPDSWDDSYVWNCDHYAVRPAIGSLQENLRQLHHHHHHHH |
| K8 |  | IHNITSFIGSVCPDGFNWGYGCAAGSSRFCTRHDWCCYDERADSHTYGFCTGNRVTGSLQENLRQLHHHHHHHH |
| K57 |  | IHNITSFIGSSGCPPGYKSGVDCSPGSECKWGCYAVDGRRYGGYGADSGVGSTGSLQENLRQLHHHHHHHH |
| aIL2_1 | Anti-parallel coiled-coil stalk from human Rabenosyn-5; PDB 1Z0J, residues 739-759 & 765-776 | LLQQIDNIKAYIGSSCPESYRFHSDRWSRNCCIPDSWDDSYVWNCDHYAVRPAIGSLTENLRELKHTLHHHHHHHH |
| K8 |  | LLQQIDNIKAYIGSVCPDGFNWGYGCAAGSSRFCTRHDWCCYDERADSHTYGFCTGNRVTGSLTENLRELKHTLHHHHHHHH |
| K57 |  | LLQQIDNIKAYIGSSGCPPGYKSGVDCSPGSECKWGCYAVDGRRYGGYGADSGVGSTGSLTENLRELKHTLHHHHHHHH |
| aIL2_1 | Anti-parallel coiled-coil stalk from human PATJ; PDB 1VF6, residues 50-67 & 50-67 | ILTLQQSIKQLKGQLNHIGSSCPESYRFHSDRWSRNCCIPDSWDDSYVWNCDHYAVRPAIGSILTLQQSIKQLKGQLNHIHHHHHHHH |
| K8 |  | ILTLQQSIKQLKGQLNHIGSVCPDGFNWGYGCAAGSSRFCTRHDWCCYDERADSHTYGFCTGNRVTGSILTLQQSIKQLKGQLNHIHHHHHHHH |
| K57 |  | ILTLQQSIKQLKGQLNHIGSSGCPPGYKSGVDCSPGSECKWGCYAVDGRRYGGYGADSGVGSTGSILTLQQSIKQLKGQLNHIHHHHHHHH |
| aIL2_1 | Anti-parallel coiled-coil stalk from human PATJ; PDB 1VF6, residues 57-67 & 50-60 | IKQLKGQLNHIGSSCPESYRFHSDRWSRNCCIPDSWDDSYVWNCDHYAVRPAIGSILTLQQSIKQLHHHHHHHH |
| K8 |  | IKQLKGQLNHIGSVCPDGFNWGYGCAAGSSRFCTRHDWCCYDERADSHTYGFCTGNRVTGSILTLQQSIKQLHHHHHHHH |
| K57 |  | IKQLKGQLNHIGSSGCPPGYKSGVDCSPGSECKWGCYAVDGRRYGGYGADSGVGSTGSILTLQQSIKQLHHHHHHHH |
| aIL2_1 | Anti-parallel coiled-coil stalk from human Beclin-1; UniProt Q14457, residues 229-247 & 194-212 | YQREYSEFKRQQLELDDELGSSCPESYRFHSDRWSRNCCIPDSWDDSYVWNCDHYAVRPAIGSLIQELEDVEKNRKIVAENLHHHHHHHH |
| K8 |  | YQREYSEFKRQQLELDDELGSVCPDGFNWGYGCAAGSSRFCTRHDWCCYDERADSHTYGFCTGNRVTGSLIQELEDVEKNRKIVAENLHHHHHHHH |
| K57 |  | YQREYSEFKRQQLELDDELGSSGCPPGYKSGVDCSPGSECKWGCYAVDGRRYGGYGADSGVGSTGSLIQELEDVEKNRKIVAENLHHHHHHHH |
| aIL2_1 | Full-length native stalk + human immunoglobulin κ light chain; UniProt P0DOX7, residues 1-3 | DIQTTVHQSTRTRESCPESYRFHSDRWSRNCCIPDSWDDSYVWNCDHYAVRPAISAYTYENHVDHHHHHHHH |
| K8 |  | DIQQQKTHQVCPDGFNWGYGCAAGSSRFCTRHDWCCYDERADSHTYGFCTGNRVTNTYEFHADHHHHHHHH |
| K57 |  | DIQTVHQRTIKSGCPPGYKSGVDCSPGSECKWGCYAVDGRRYGGYGADSGVGSTYTHEFYVDHHHHHHHH |
| aIL2_1 | Shortened native stalk + human immunoglobulin κ light chain; UniProt P0DOX7, residues 1-3 | DIQSTRTRESCPESYRFHSDRWSRNCCIPDSWDDSYVWNCDHYAVRPAISAYTYEHHHHHHHH |
| K8 |  | DIQKTHQVCPDGFNWGYGCAAGSSRFCTRHDWCCYDERADSHTYGFCTGNRVTNTYEHHHHHHHH |
| K57 |  | DIQRTIKSGCPPGYKSGVDCSPGSECKWGCYAVDGRRYGGYGADSGVGSTYTHEHHHHHHHH |
| aIL2_1 | Human immunoglobulin κ light chain; UniProt P0DOX7, residues 1-3 | DIQSCPESYRFHSDRWSRNCCIPDSWDDSYVWNCDHYAVRPAIHHHHHHHH |
| K8 |  | DIQVCPDGFNWGYGCAAGSSRFCTRHDWCCYDERADSHTYGFCTGNRVTHHHHHHHH |
| K57 |  | DIQSGCPPGYKSGVDCSPGSECKWGCYAVDGRRYGGYGADSGVGSTHHHHHHHH |
| aIL2_1 | Human immunoglobulin κ light chain; UniProt P0DOX7, residues 1-3 with GGGGS-linker | DIQGGGGSSCPESYRFHSDRWSRNCCIPDSWDDSYVWNCDHYAVRPAIHHHHHHHH |
| K8 |  | DIQGGGGSVCPDGFNWGYGCAAGSSRFCTRHDWCCYDERADSHTYGFCTGNRVTHHHHHHHH |
| K57 |  | DIQGGGGSSGCPPGYKSGVDCSPGSECKWGCYAVDGRRYGGYGADSGVGSTHHHHHHHH |
| aIL2_1 | Human immunoglobulin κ light chain; UniProt P0DOX7, residues 1-7 with GGGGS-linker | DIQMTQSGGGGSSCPESYRFHSDRWSRNCCIPDSWDDSYVWNCDHYAVRPAIHHHHHHHH |
| K8 |  | DIQMTQSGGGGSVCPDGFNWGYGCAAGSSRFCTRHDWCCYDERADSHTYGFCTGNRVTHHHHHHHH |
| K57 |  | DIQMTQSGGGGSSGCPPGYKSGVDCSPGSECKWGCYAVDGRRYGGYGADSGVGSTHHHHHHHH |
| aIL2_1 | Human immunoglobulin γ heavy chain; UniProt P0DOX5, residues 1-4 with GS-linker | QVQLGSSCPESYRFHSDRWSRNCCIPDSWDDSYVWNCDHYAVRPAIHHHHHHHH |
| K8 |  | QVQLGSVCPDGFNWGYGCAAGSSRFCTRHDWCCYDERADSHTYGFCTGNRVTHHHHHHHH |
| K57 |  | QVQLGSSGCPPGYKSGVDCSPGSECKWGCYAVDGRRYGGYGADSGVGSTHHHHHHHH |
| aIL2_1 | Human immunoglobulin γ heavy chain; UniProt P0DOX5, residues 1-8 with GS-linker | QVQLVQSGSSCPESYRFHSDRWSRNCCIPDSWDDSYVWNCDHYAVRPAIHHHHHHHH |
| K8 |  | QVQLVQSGSVCPDGFNWGYGCAAGSSRFCTRHDWCCYDERADSHTYGFCTGNRVTHHHHHHHH |
| K57 |  | QVQLVQSGSSGCPPGYKSGVDCSPGSECKWGCYAVDGRRYGGYGADSGVGSTHHHHHHHH |
| aIL2_1 | Anti-parallel coiled-coil stalk from *Sin Nombre orthohantavirus* nucleocapsid protein; PDB 2IC6, residues 4-11 & 61-68 + human immunoglobulin κ light chain; UniProt P0DOX7, residues 1-3 | DIQLKEVQDNIGSSCPESYRFHSDRWSRNCCIPDSWDDSYVWNCDHYAVRPAIGSLKRELADLHHHHHHHH |
| K8 |  | DIQLKEVQDNIGSVCPDGFNWGYGCAAGSSRFCTRHDWCCYDERADSHTYGFCTGNRVTGSLKRELADLHHHHHHHH |
| K57 |  | DIQLKEVQDNIGSSGCPPGYKSGVDCSPGSECKWGCYAVDGRRYGGYGADSGVGSTGSLKRELADLHHHHHHHH |
| aIL2_1 | N-terminal α-helix from human haemoglobin B; UniProt P68871, residues 2-16 | VHLTPEEKSAVTALWGSSCPESYRFHSDRWSRNCCIPDSWDDSYVWNCDHYAVRPAIHHHHHHHH |
| K8 |  | VHLTPEEKSAVTALWGSVCPDGFNWGYGCAAGSSRFCTRHDWCCYDERADSHTYGFCTGNRVTHHHHHHHH |
| K57 |  | VHLTPEEKSAVTALWGSSGCPPGYKSGVDCSPGSECKWGCYAVDGRRYGGYGADSGVGSTHHHHHHHH |
| aIL2_1 | N-terminal α -helix from human serum albumin; UniProt P02768, residues 25-37 | DAHKSEVAHRFKDGSSCPESYRFHSDRWSRNCCIPDSWDDSYVWNCDHYAVRPAIHHHHHHHH |
| K8 |  | DAHKSEVAHRFKDGSVCPDGFNWGYGCAAGSSRFCTRHDWCCYDERADSHTYGFCTGNRVTHHHHHHHH |
| K57 |  | DAHKSEVAHRFKDGSSGCPPGYKSGVDCSPGSECKWGCYAVDGRRYGGYGADSGVGSTHHHHHHHH |
| aIL2_1 | N- and C-terminal α-helices from human serum albumin; UniProt P02768, residues 25-37 & 597-609 | DAHKSEVAHRFKDGSSCPESYRFHSDRWSRNCCIPDSWDDSYVWNCDHYAVRPAIGSKKLVAASQAALGLHHHHHHHH |
| K8 |  | DAHKSEVAHRFKDGSVCPDGFNWGYGCAAGSSRFCTRHDWCCYDERADSHTYGFCTGNRVTGSKKLVAASQAALGLHHHHHHHH |
| K57 |  | DAHKSEVAHRFKDGSSGCPPGYKSGVDCSPGSECKWGCYAVDGRRYGGYGADSGVGSTGSKKLVAASQAALGLHHHHHHHH |


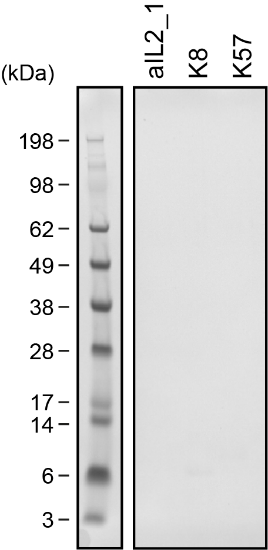


**Supplementary Figure S2.** SDS-PAGE analysis of Ni-IMAC eluates showing negligible recombinant expression levels of knob domains with native β-ribbon stalks. The gels were run under reducing conditions. Theoretical molecular weights of the analysed constructs are 7.5-8.5 kDa.


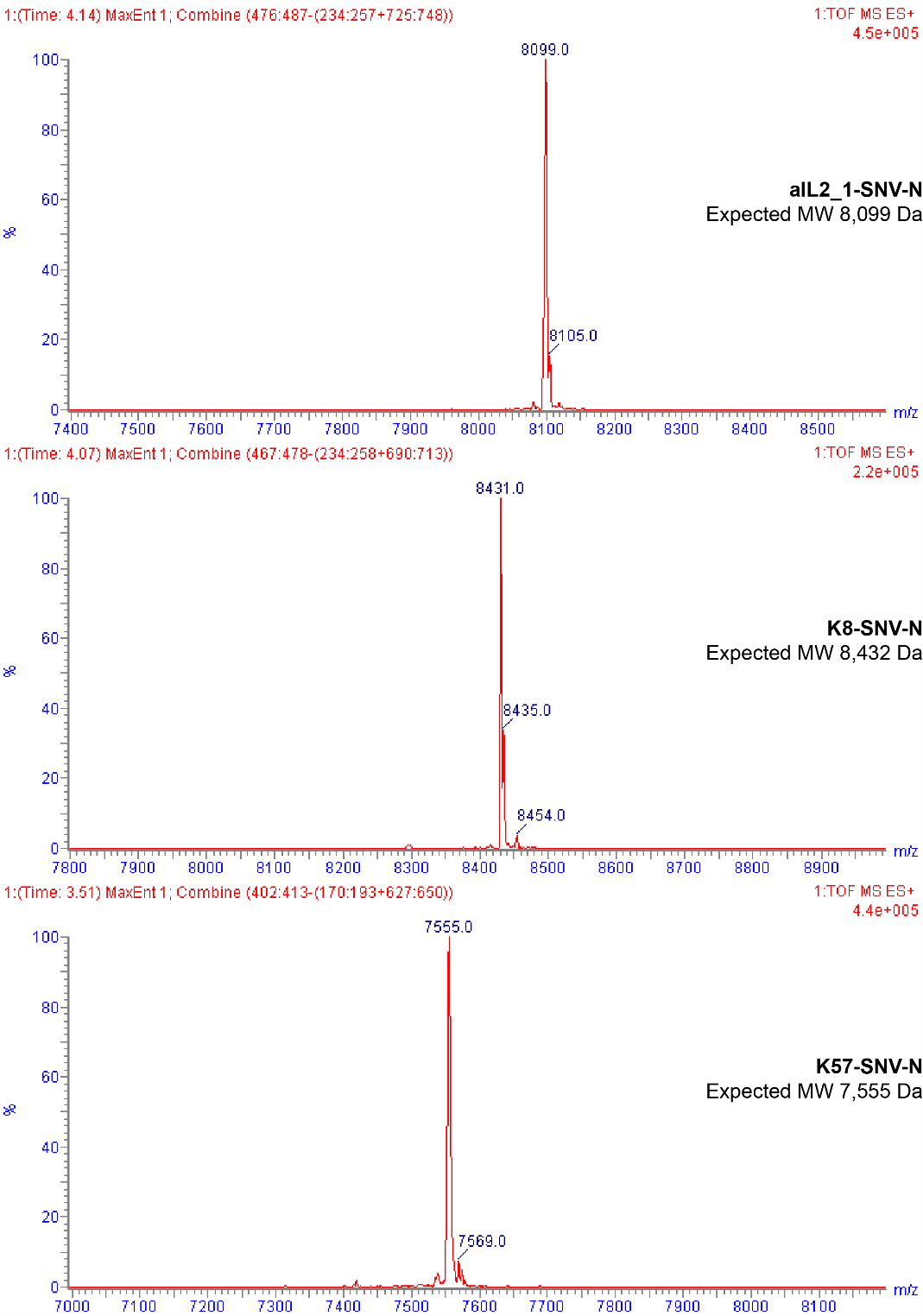


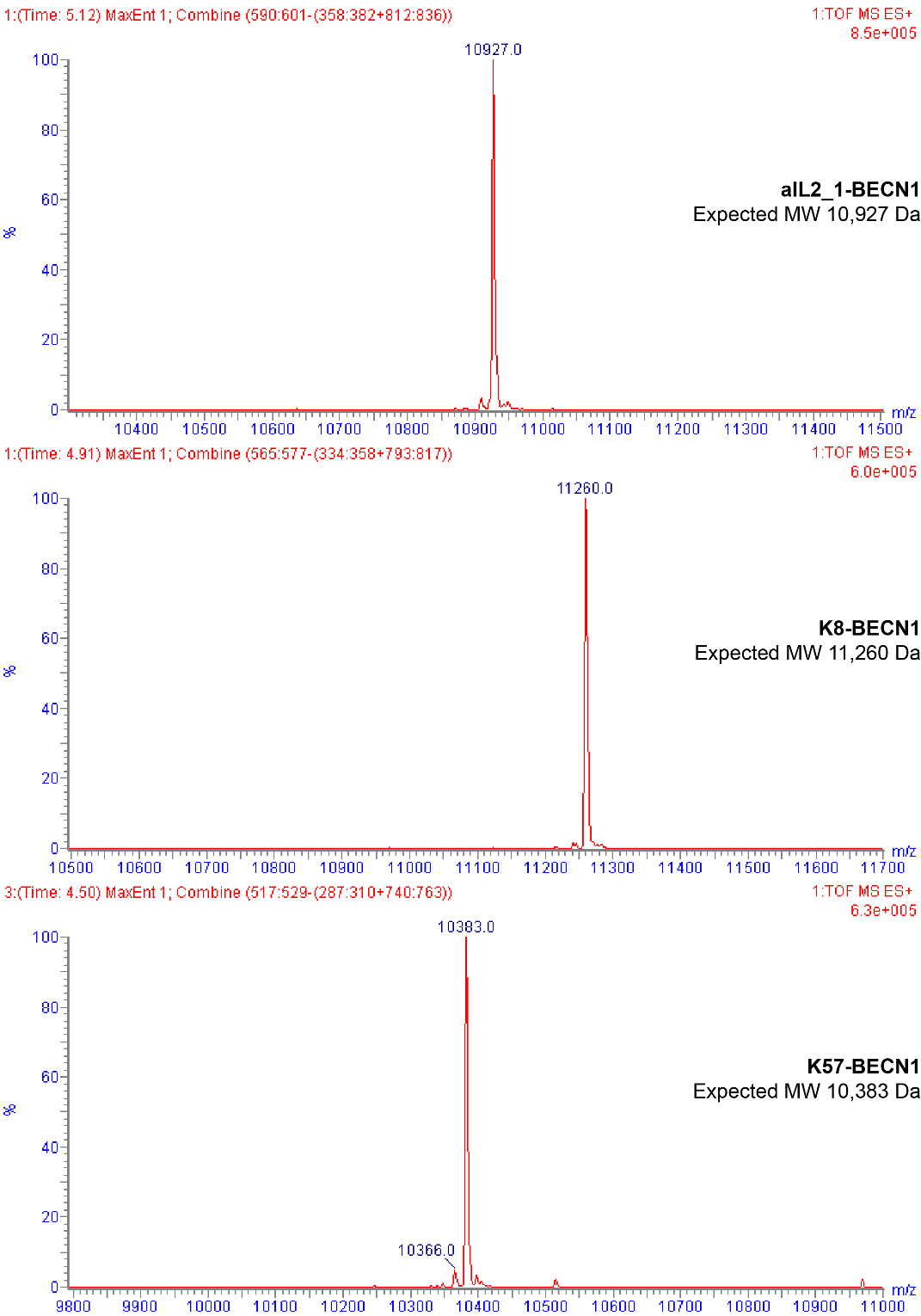


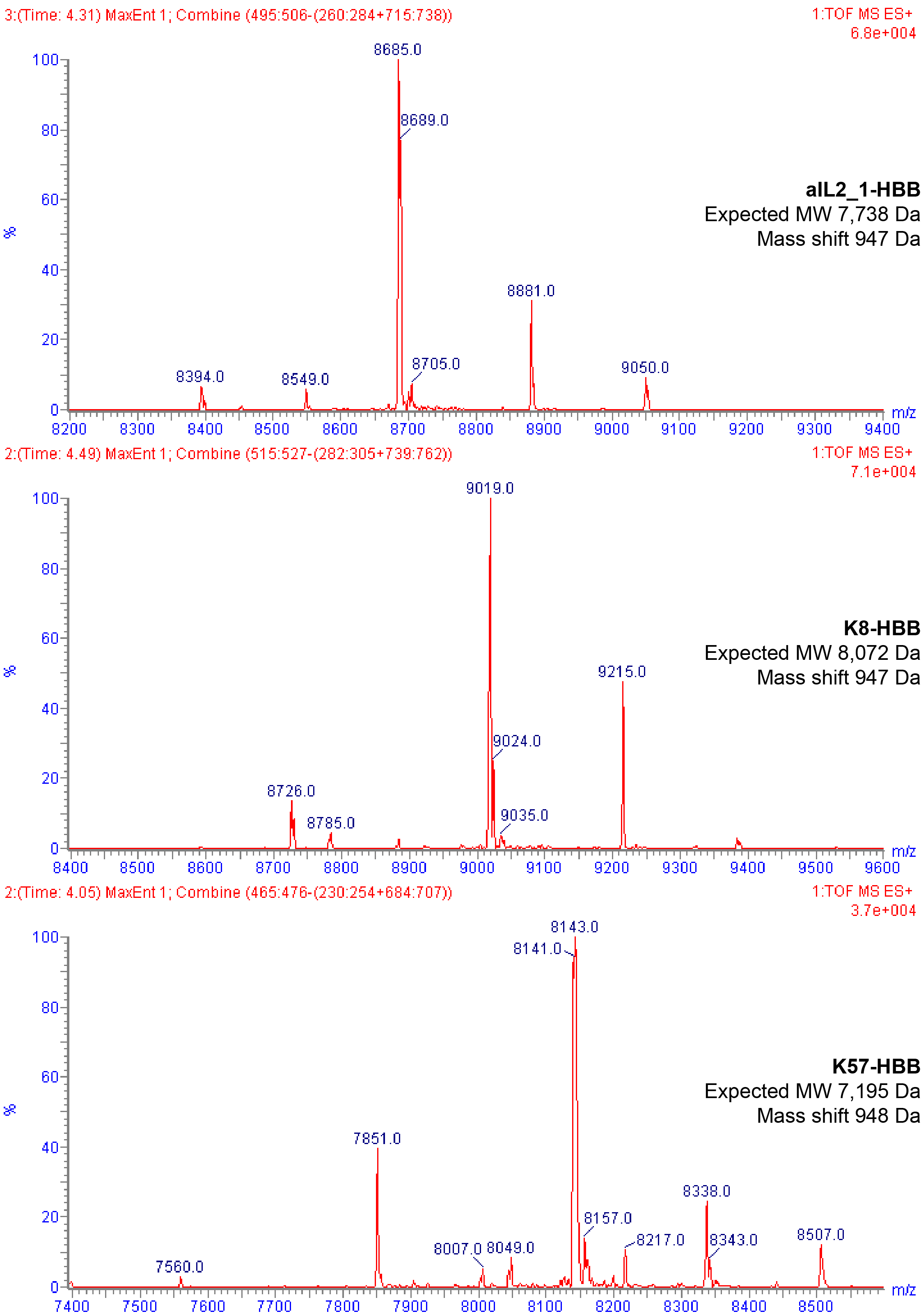


**Supplementary Figure S3.** Representative mass spectra of knob domain expression constructs. A shift of 948 Da is indicative of the presence of an *O*-linked tetrasaccharide -GalNAc(-NeuNAc)-Gal-NeuNAc, which is commonly observed in recombinant proteins produced by HEK cells.


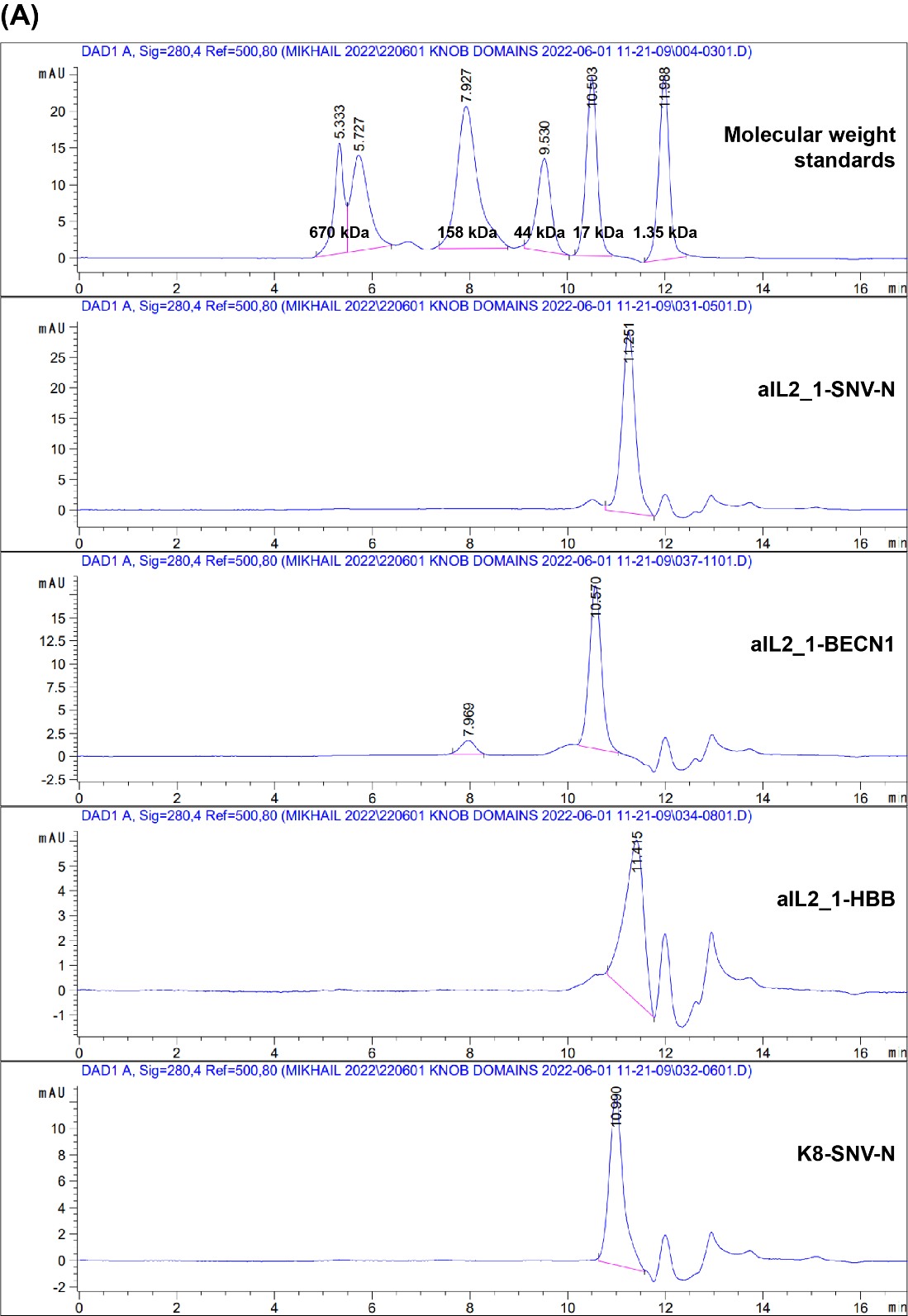


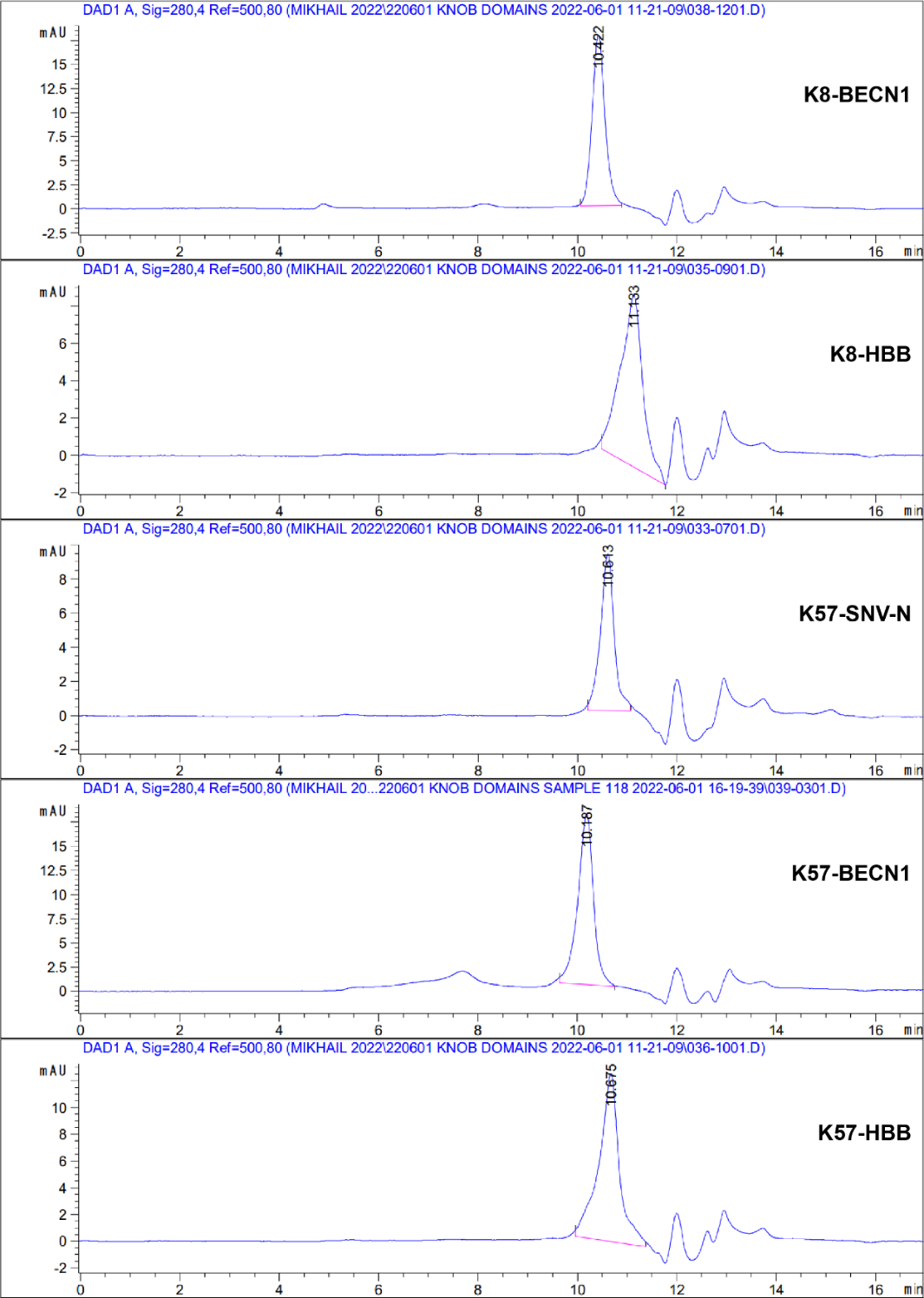


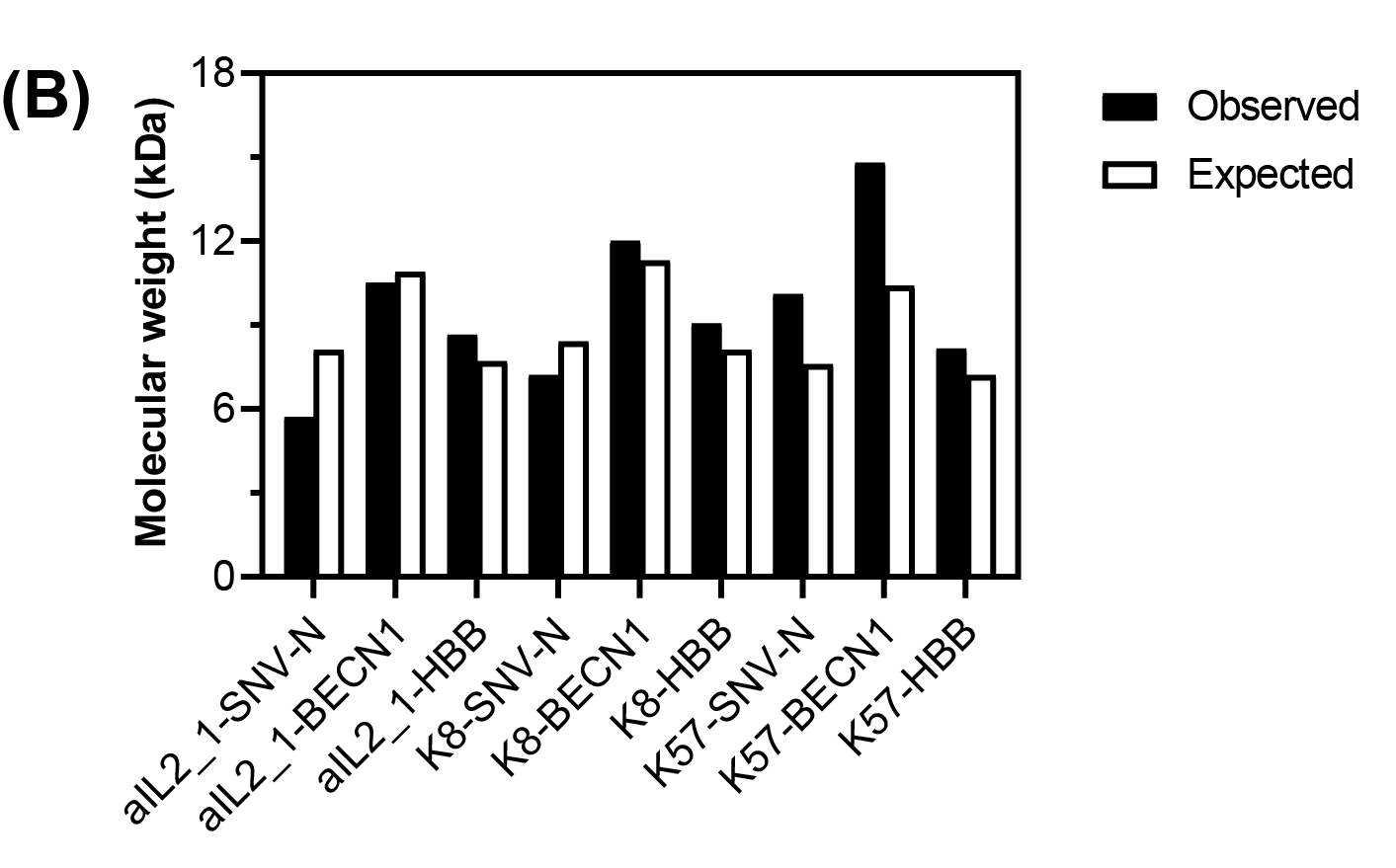


**Supplementary Figure S4.** SEC-HPLC analysis of knob domain expression constructs. **(A)** Representative chromatograms. **(B)** Comparison of predicted monomeric molecular weights with the observed data.


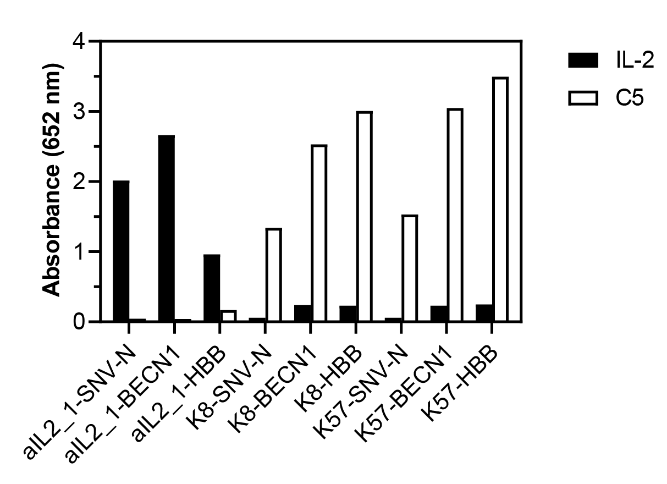


**Supplementary Figure S5.** Antigen binding of SNV-N-, BECN1- and HBB-fused knob domains assessed by ELISA. ELISA plates were coated with IL-2 or C5, incubated with 1:5000 (aIL2_1) or 1:500 (K8 and K57) of IMAC-enriched cell culture media and developed using anti-His as detection antibodies. Uncoated wells were used as a negative control.


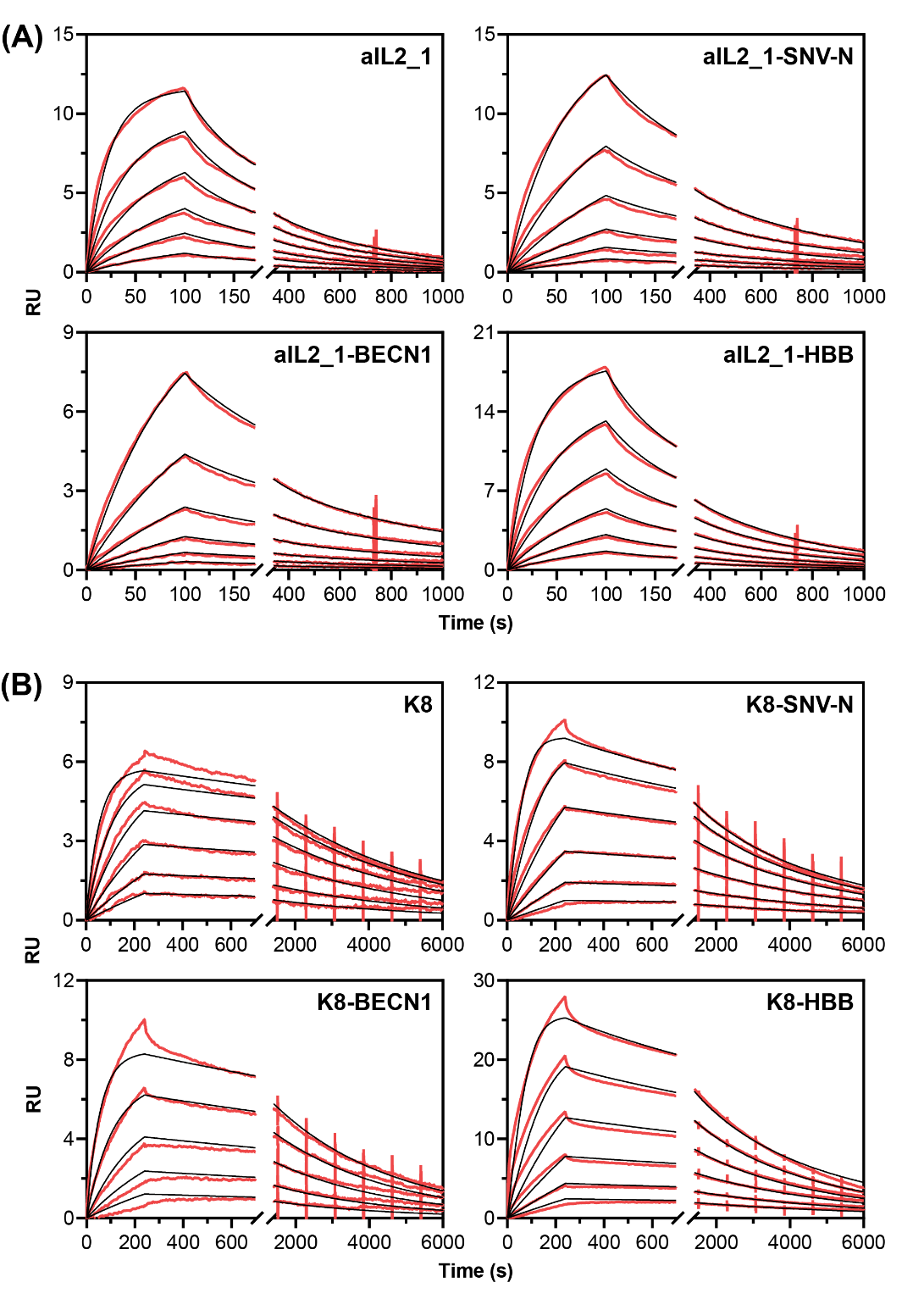


**Supplementary Figure S6.** Representative sensorgrams for binding of aIL2_1 **(A)** and K8 **(B)** to their targets. Knob domains alone were synthetised by SPPS; the knob domain fusions were recombinantly produced in mammalian cells. Immobilised antigens were subject to the injections of various concentrations of knob domains. Experimental data are shown in red; the lines of best fit are shown in black.


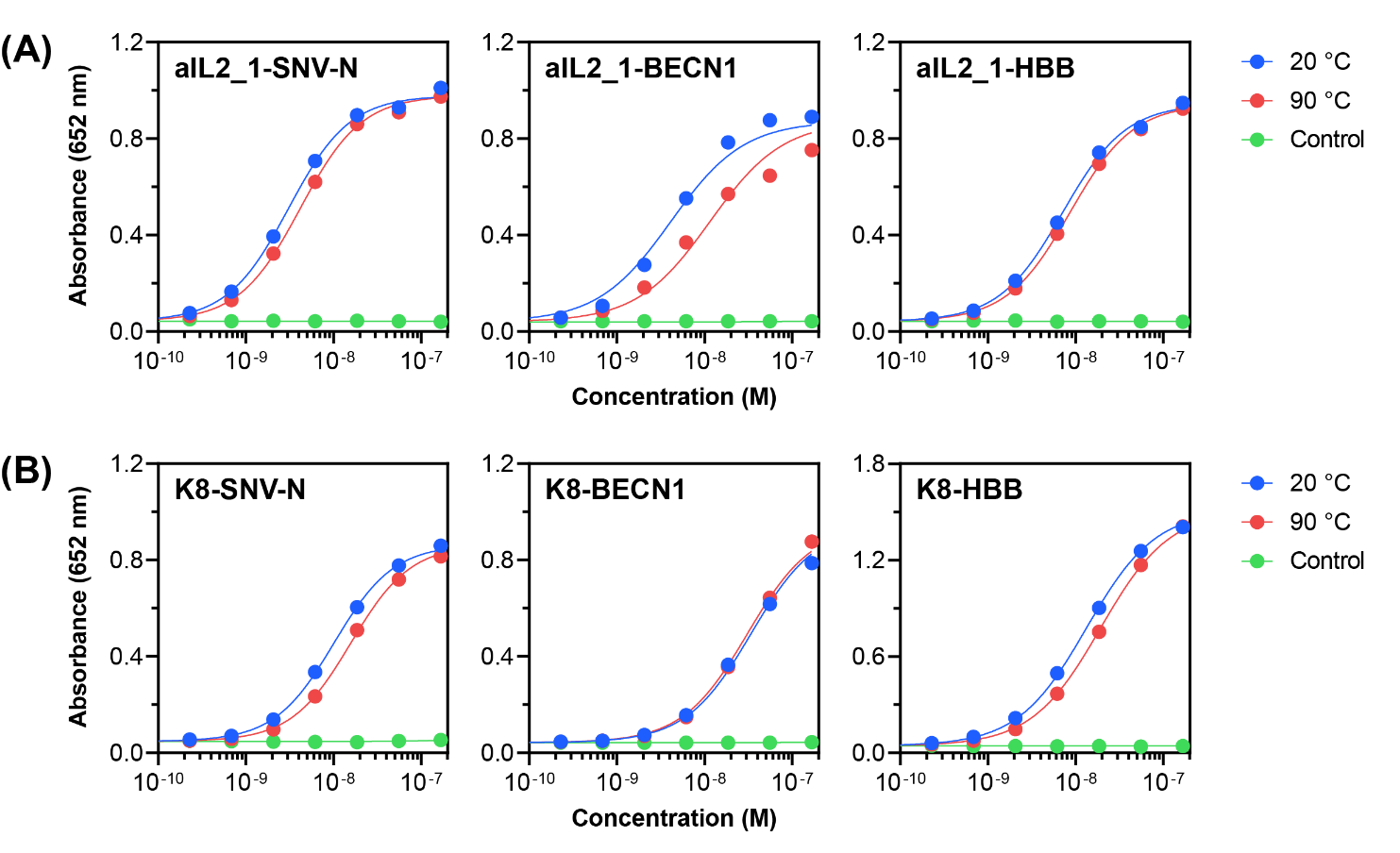


**Supplementary Figure S7.** Antigen-binding activity of aIL2_1 **(A)** and K8 **(B)** fusion constructs after a thermal stress. The proteins were kept at 20 °C or heated to 90 °C for 30 min and analysed by ELISA; uncoated blocked wells treated with 20 °C samples served as negative control.


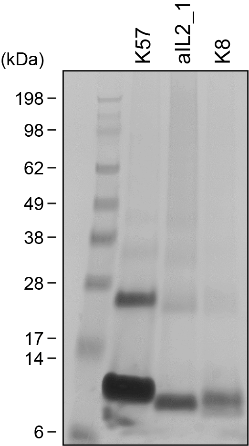


**Supplementary Figure S8.** SDS-PAGE analysis of BECN1-fused knob domains expressed in bacterial cells. The gels were run under non-reducing conditions. Theoretical molecular weights of monomeric fusions are 10-11 kDa.


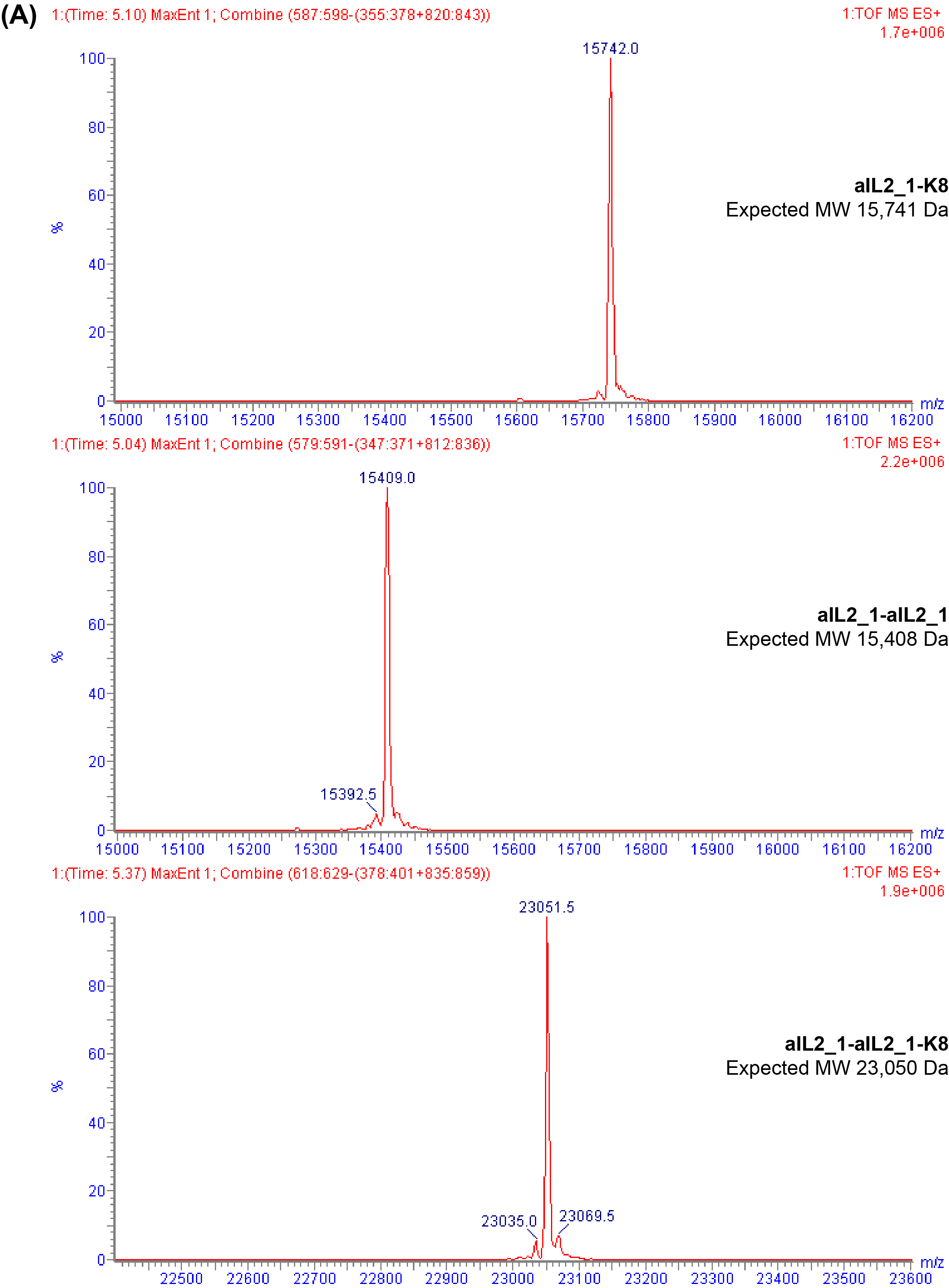


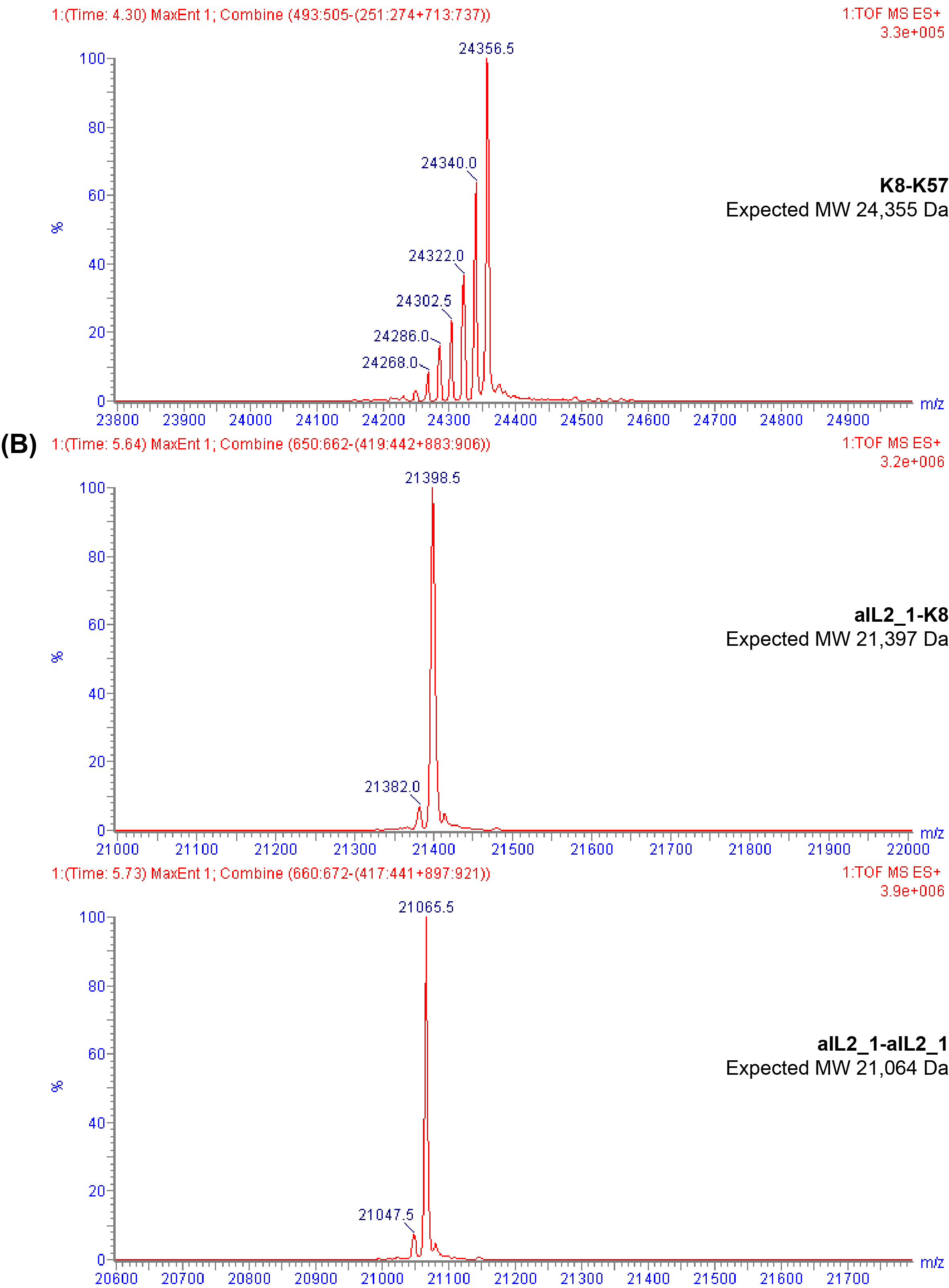


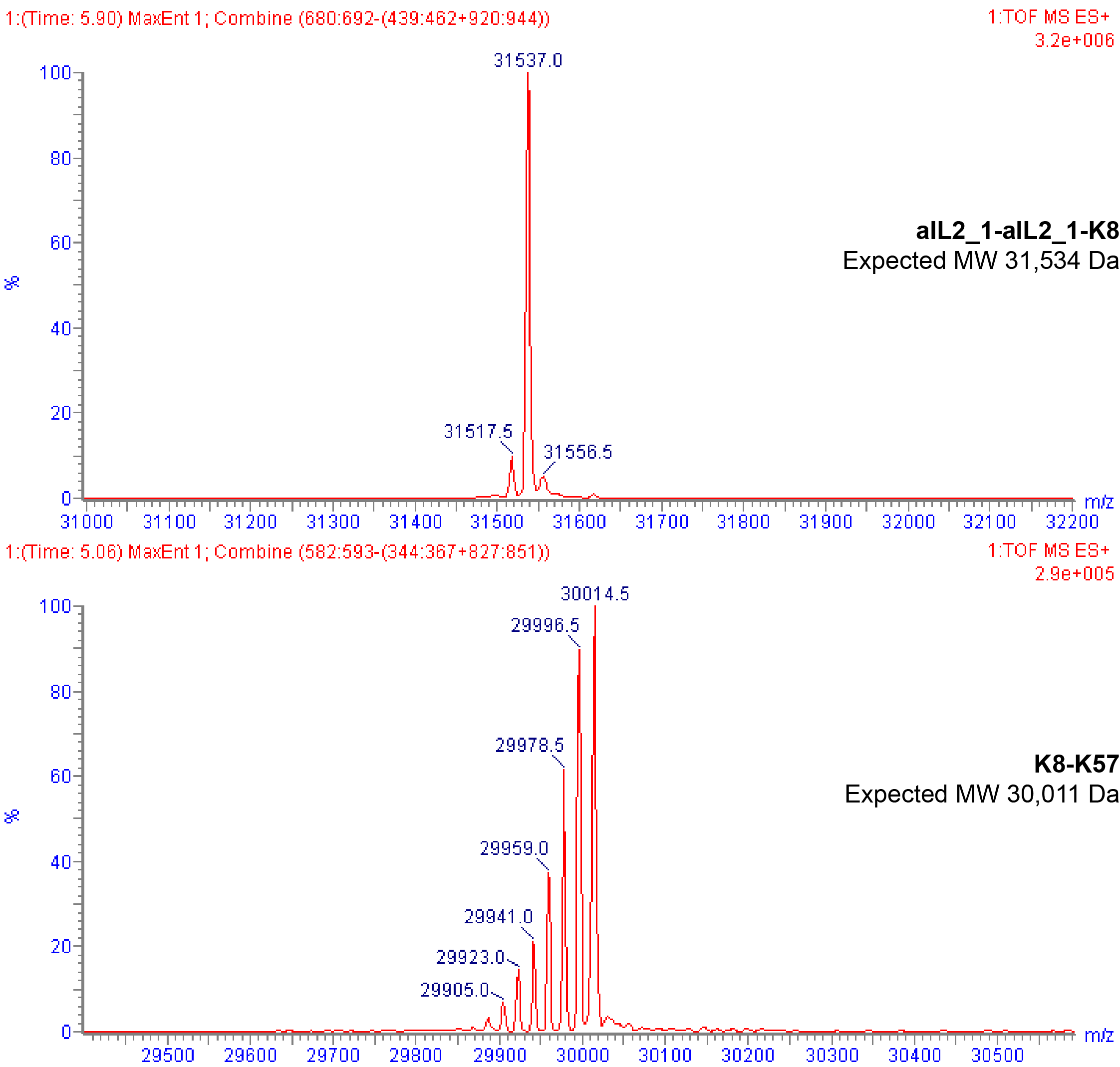


**Supplementary Figure S9.** Representative mass spectra of bispecific knob domains constructs. The knob domains were fused to SNV-N **(A)** or BECN1 **(B)** stalks.


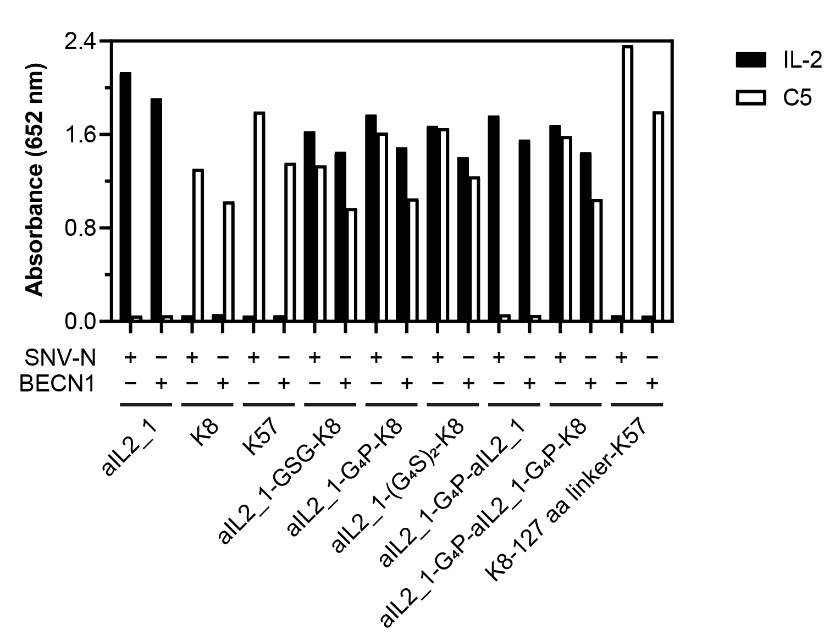


**Supplementary Figure S10.** Antigen binding of bispecific, bivalent and biparatopic knob domains assessed by ELISA. ELISA plates were coated with IL-2 or C5, incubated with 1:2700 of IMAC-enriched cell culture media and developed using anti-His as detection antibodies. Individual aIL2_1, K8 and K57 knob domains were used as controls.

**Supplementary Table S11.** Antigen-binding kinetics of bispecific, bivalent and biparatopic knob domain fusions.

| Construct | Frameworks | *k*_a_, M^-1^s^-1^ | *k*_d_, s^-1^ | *K*_D_, M |
| --- | --- | --- | --- | --- |
| Knob domains immobilised on the surface, C5 in solution | | | | |
| K8 | SNV-N | 8.4 × 10^3^ | 3.6 × 10^-4^ | 4.3 × 10^-8^ |
|  | BECN1 | 7.2 × 10^3^ | 4.3 × 10^-4^ | 6.1 × 10^-8^ |
| K57 | SNV-N | 1.12 × 10^4^ | 3.6 × 10^-4^ | 3.2 × 10^-8^ |
|  | BECN1 | 8.1 × 10^3^ | 4.2 × 10^-4^ | 5.2 × 10^-8^ |
| aIL2_1-K8 (G_4_P linker) | SNV-N | 9.7 × 10^3^ | 2.5 × 10^-4^ | 2.6 × 10^-8^ |
|  | BECN1 | 8.8 × 10^3^ | 2.6 × 10^-4^ | 2.9 × 10^-8^ |
| K8-K57 | SNV-N | 1.54 × 10^4^ | 1.83 × 10^-5^ | 1.18 × 10^-9^ |
|  | BECN1 | 3.1 × 10^4^ | 2.8 × 10^-5^ | 9.0 × 10^-10^ |
| Knob domains immobilised on the surface, IL-2 in solution | | | | |
| aIL2_1 | SNV-N | 6.6 × 10^5^ | 1.33 × 10^-3^ | 9.4 × 10^-9^ |
|  | BECN1 | 5.2 × 10^5^ | 1.31 × 10^-3^ | 1.08 × 10^-8^ |
| aIL2_1-K8 (G_4_P linker) | SNV-N | 4.7 × 10^5^ | 1.31 × 10^-3^ | 9.0 × 10^-9^ |
|  | BECN1 | 4.7 × 10^5^ | 1.34 × 10^-3^ | 1.00 × 10^-8^ |
| aIL2_1-aIL2_1 | SNV-N | 3.4 × 10^5^ | 9.8 × 10^-4^ | 5.7 × 10^-9^ |
|  | BECN1 | 3.5 × 10^5^ | 1.08 × 10^-3^ | 7.5 × 10^-9^ |
| aIL2_1-aIL2_1-K8 | SNV-N | 2.5 × 10^5^ | 9.2 × 10^-4^ | 5.5 × 10^-9^ |
|  | BECN1 | 3.2 × 10^5^ | 1.09 × 10^-3^ | 7.1 × 10^-9^ |
| IL-2 immobilised on the surface, knob domains in solution | | | | |
| aIL2_1 | SNV-N | 2.3 × 10^5^ | 2.5 × 10^-3^ | 1.79 × 10^-8^ |
|  | BECN1 | 1.43 × 10^5^ | 1.85 × 10^-3^ | 2.3 × 10^-8^ |
| aIL2_1-aIL2_1 | SNV-N | 6.7 × 10^5^ | 4.8 × 10^-4^ | 3.6 × 10^-10^ |
|  | BECN1 | 5.1 × 10^4^ | 2.7 × 10^-4^ | 7.9 × 10^-10^ |
| aIL2_1-aIL2_1-K8 | SNV-N | 1.47 × 10^5^ | 5.1 × 10^-4^ | 7.9 × 10^-10^ |
|  | BECN1 | 4.4 × 10^4^ | 1.21 × 10^-5^ | 4.8 × 10^-11^ |


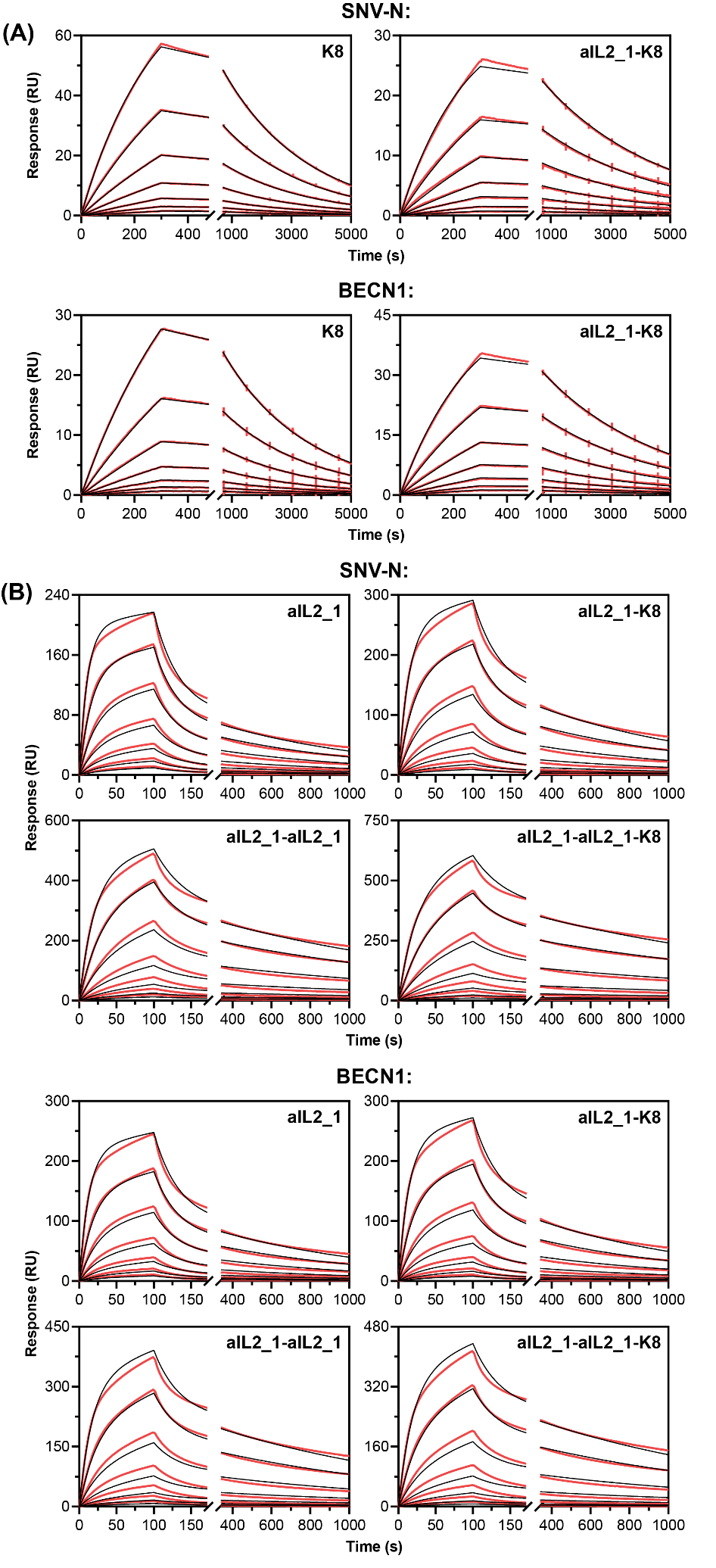


**Supplementary Figure S12.** Representative sensorgrams for binding of individual knob domains and bispecific fusions to their targets. Immobilised knob domains were subject to the injections of various concentrations of human C5 **(A)** and human IL-2 **(B)**. Experimental data are shown in red; the lines of best fit are shown in black.


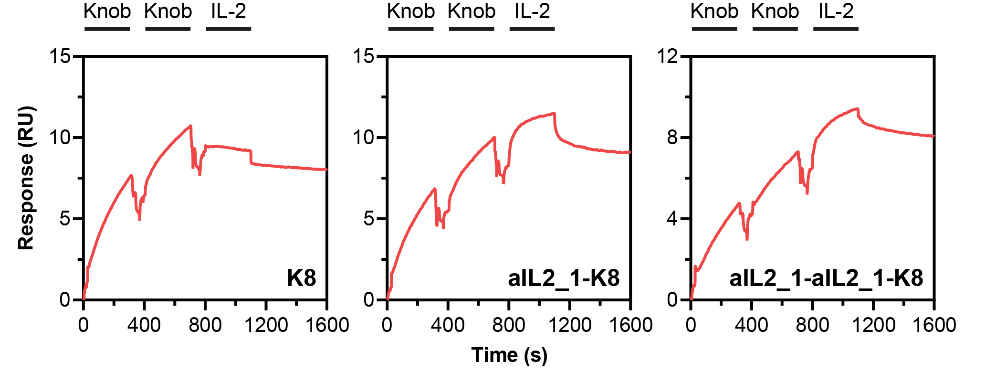


**Supplementary Figure S13.** The bridging SPR assay confirms simultaneous binding of BECN1-fused bispecific knob domain fusions to their antigens. The surface was coated with C5 followed by the addition of bispecific fusion. IL-2 was then added. Dissociation of the complex is measured by washing the surface with buffer.


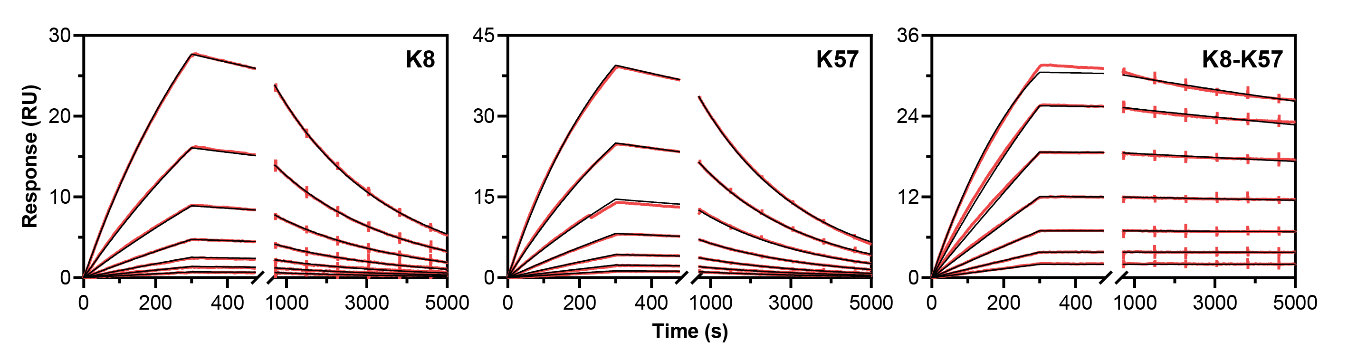


**Supplementary Figure S14.** Representative sensorgrams for binding of an anti-C5 biparatopic K8-K57 knob domain fusion to C5. The knob domain stalks were substituted with BECN1 helices. Individual K8 and K57 were used as controls. Immobilised knob domains were subject to the injections of various concentrations of C5. Experimental data are shown in red; the lines of best fit are shown in black.


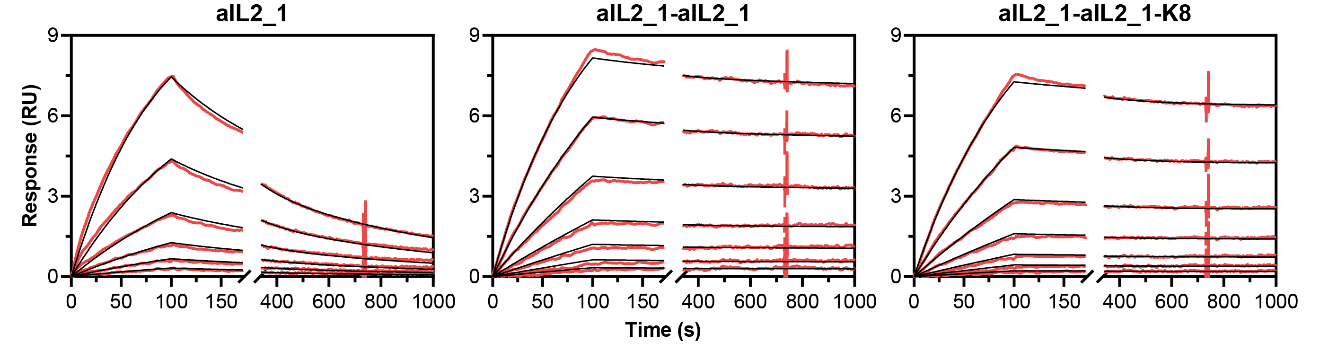


**Supplementary Figure S15.** Representative sensorgrams for binding of anti-IL-2 bivalent knob domain fusions to IL-2. The knob domain stalks were substituted with BECN1 helices. Individual aIL2_1 was used as control. Immobilised IL-2 was subject to the injections of various concentrations of knob domains. Experimental data are shown in red; the lines of best fit are shown in black.
